# Effects of acute cannabidiol on behavior and the endocannabinoid system in HIV-1 Tat transgenic female and male mice

**DOI:** 10.3389/fnins.2024.1358555

**Published:** 2024-03-05

**Authors:** Barkha J. Yadav-Samudrala, Benjamin L. Gorman, Karenna M. Barmada, Havilah P. Ravula, Caitlin J. Huguely, E. Diane Wallace, Michelle R. Peace, Justin L. Poklis, Wei Jiang, Sylvia Fitting

**Affiliations:** ^1^Department of Psychology and Neuroscience, University of North Carolina at Chapel Hill, Chapel Hill, NC, United States; ^2^Department of Chemistry, University of North Carolina at Chapel Hill, Chapel Hill, NC, United States; ^3^Department of Pharmacology and Toxicology, Virginia Commonwealth University, Richmond, VA, United States; ^4^Department of Microbiology and Immunology, Medical University of South Carolina, Charleston, SC, United States; ^5^Division of Infectious Diseases, Department of Medicine, Medical University of South Carolina, Charleston, SC, United States

**Keywords:** cannabidiol, Tat transgenic mice, antinociception, 2-arachidonoylglycerol, arachidonic acid, GPR55, FAAH

## Abstract

**Background:**

Some evidence suggests that cannabidiol (CBD) has potential to help alleviate HIV symptoms due to its antioxidant and anti-inflammatory properties. Here we examined acute CBD effects on various behaviors and the endocannabinoid system in HIV Tat transgenic mice.

**Methods:**

Tat transgenic mice (female/male) were injected with CBD (3, 10, 30 mg/kg) and assessed for antinociception, activity, coordination, anxiety-like behavior, and recognition memory. Brains were taken to quantify endocannabinoids, cannabinoid receptors, and cannabinoid catabolic enzymes. Additionally, CBD and metabolite 7-hydroxy-CBD were quantified in the plasma and cortex.

**Results:**

Tat decreased supraspinal-related nociception and locomotion. CBD and sex had little to no effects on any of the behavioral measures. For the endocannabinoid system male sex was associated with elevated concentration of the proinflammatory metabolite arachidonic acid in various CNS regions, including the cerebellum that also showed higher FAAH expression levels for Tat(+) males. GPR55 expression levels in the striatum and cerebellum were higher for females compared to males. CBD metabolism was altered by sex and Tat expression.

**Conclusion:**

Findings indicate that acute CBD effects are not altered by HIV Tat, and acute CBD has no to minimal effects on behavior and the endocannabinoid system.

## Introduction

In 2022, 39 million people were living with human immunodeficiency virus (HIV) worldwide, out of which ~75% have access to antiretroviral therapy (ART; UNAIDS, 2023). While combination ART (cART) has significantly increased survival rates and quality of life (May et al., 2006; Harrison et al., 2010; Marcus et al., 2016), 30%–50% of cART-treated people living with HIV (PLWH) exhibit some degree of cognitive deficits, known as HIV-associated neurocognitive disorders (HAND; Ellis et al., 2007; Heaton et al., 2010; Sacktor et al., 2016; Ajasin and Eugenin, 2020). Typical HAND symptoms include deficits in attention, learning and memory, and slowed motor control (Antinori et al., 2007; Ellis et al., 2007; Thames et al., 2016, 2017), which can lead to difficulty with activities of daily living (Heaton et al., 1995, 2004; Marcotte et al., 2004; Ellis et al., 2007) as well as unemployment (Heaton et al., 1994; Albert et al., 1995; van Gorp et al., 1999). Additional central nervous system (CNS)-related symptoms reported by PLWH include neuropathic pain (Martin et al., 2003) and heightened anxiety (Whetten et al., 2008; Mitra and Sharman, 2022).

HAND and HIV-related CNS symptoms persist in cART-treated PLWH as a result of chronic immune activation, neuroinflammation, and low-level expression of viral proteins (Mediouni et al., 2012; Ajasin and Eugenin, 2020). One such viral protein is HIV transactivator of transcription (Tat), which is observed rapidly in the CNS after initial HIV infection (King et al., 2006; Das et al., 2011), and is particularly elevated in the brains of PLWH with HAND (Wiley et al., 1996; Hudson et al., 2000). Multiple *in vitro* and *in vivo* studies have demonstrated Tat’s role in neurotoxicity in HAND, even in the absence of any other HIV viral proteins (Carey et al., 2012; Bertrand et al., 2013; Fitting et al., 2013; Hahn et al., 2015; Wodarski et al., 2018; Zhao et al., 2020; League et al., 2021; Marks et al., 2021). HIV Tat is secreted by infected cells (Ensoli et al., 1990; Sabatier et al., 1991; Conant et al., 1994; King et al., 2006; Kannan et al., 2022) and can interact with cell surface receptors of uninfected neuronal and glial cells (Philippon et al., 1994; Prendergast et al., 2002; Fitting et al., 2014), thus directly damaging neuronal structure and function (Sabatier et al., 1991; Cheng et al., 1998; Bertrand et al., 2014; Hermes et al., 2021), as well as indirectly by promoting proinflammatory signaling via glial activation (Nath et al., 1999; Hahn et al., 2010; Jin et al., 2012). Further, using the HIV Tat transgenic mouse model, various studies have demonstrated deficits in learning and memory, motivation, and motor activity (Fitting et al., 2013; Kesby et al., 2016; Zhao et al., 2020; League et al., 2021), as well as Tat’s contribution to HIV-related neuropathic pain (Chi et al., 2011; Wodarski et al., 2018; Bagdas et al., 2020; Cirino et al., 2021) and anxiety (Paris et al., 2014; Hahn et al., 2016; Kasten et al., 2017; Joshi et al., 2020; Salahuddin et al., 2020; Qrareya et al., 2021), all of which recapitulate the pathological and behavioral abnormalities associated with HAND.

The endocannabinoid system is a potential source of neuroprotection and anti-inflammatory action (Bie et al., 2018; Yadav-Samudrala and Fitting, 2021), and cannabis use has been reported to alleviate HIV-related symptoms, such as pain, anxiety, stress, and loss of appetite (Woolridge et al., 2005; Shiau et al., 2017; Costiniuk et al., 2019; Wardell et al., 2022). The use of cannabis is highly prevalent in PLWH, with cannabis use being 2–3 times greater than use among the general US population (Whitfield et al., 1997; Cristiani et al., 2004; Pacek et al., 2018; Ellis et al., 2021). Cannabis contains exogenous modulators of the endocannabinoid system, including the principle psychoactive cannabinoid, Δ^9^-tetrahydrocannabinol (THC), and the other major cannabis constitute, cannabidiol (CBD). Whereas CBD can alter affective behavior and thus be considered psychoactive (Guimaraes et al., 1990; Long et al., 2010; Xu et al., 2019; Fabris et al., 2022; Garcia-Gutierrez et al., 2023), effects of CBD are different from THC as it does not seem to possess rewarding and addictive properties (Hay et al., 2018; Viudez-Martinez et al., 2019; Navarrete et al., 2021) or produce intoxicating effects where performance or cognition is impaired (Corroon and Phillips, 2018; Morgan et al., 2018; Dahlgren et al., 2022). Specifically, CBD has been shown to have antioxidant, anti-inflammatory, and neuroprotective properties (Iuvone et al., 2004; Booz, 2011; Hughes and Herron, 2019), and several studies have reported its potential therapeutic role in attenuating memory deficits in various disease conditions (Cheng et al., 2014; Osborne et al., 2017a,b; Garcia-Baos et al., 2021). CBD is known to interact with multiple targets within the CNS to exert its effects (Atalay et al., 2020). CBD has low binding affinity for cannabinoid type 1 and 2 receptors (CB_1_R and CB_2_R, respectively; McPartland et al., 2015), even though CBD can still exert effects mediated by these receptors (Pertwee, 2008; Sagredo et al., 2011; Laprairie et al., 2015; Vilela et al., 2017; Fogaca et al., 2018; Galaj et al., 2020). A variety of other non-cannabinoid receptors can be targeted by CBD, including transient receptor potential vanilloid (TRPV) channels (Hassan et al., 2014), serotonin (5-HT1A) receptors (Resstel et al., 2009; Zanelati et al., 2010), peroxisome proliferator-activated receptor gamma (*PPARγ*; Esposito et al., 2011; Sonego et al., 2021; Khosropoor et al., 2023), and the cannabinoid-related receptor GPR55 (Ryberg et al., 2007; Burstein, 2015). For example, improvement of object recognition memory deficits in antipsychotic mice by CBD was associated with decreases in proinflammatory cytokine levels in the hippocampus, both of which were mediated by *PPARγ* receptors (Sonego et al., 2021). On the other hand, anxiolytic and neuroprotective effects of CBD in chronically stressed mice were reported to be related to CB_1_R and CB_2_R activation (Campos et al., 2013; Fogaca et al., 2018) due to upregulation of hippocampal endocannabinoid *N*-arachidonoylethanolamine (AEA) concentration via fatty acid amide hydrolase (FAAH) inhibition (Bisogno et al., 2001; Campos et al., 2013; Boggs et al., 2018).

In the present study we used HIV-1 Tat transgenic mice to investigate the acute effects of CBD (3, 10, 30 mg/kg) in neuroHIV on multiple behavioral outcomes, including pain sensitivity, motor activity, motor coordination, anxiety, and object recognition memory. Further, acute CBD effects on the endocannabinoid and endocannabinoid-like (“paracannabinoid”) systems in the CNS, including the prefrontal cortex, striatum, cerebellum, and spinal cord, were assessed by quantifying concentration of AEA, 2-arachidonoylglycerol (2-AG), *N*-palmitoylethanolamide (PEA), *N*-oleoylethanolamide (OEA), and their proinflammatory metabolite arachidonic acid (AA) via ultraperformance liquid chromatography/tandem mass spectrometry (UPLC-MS/MS). Additionally, using Western blot analyses, protein expression levels of cannabinoid receptors and endocannabinoid degradative enzymes were assessed in the striatum and cerebellum. CBD metabolism was examined via UPLC-MS/MS by quantifying concentration of CBD and its main oxidative metabolite 7-hydroxy-CBD concentration (COOH-CBD) in the plasma and cortex of acute CBD-exposed Tat transgenic mice.

## Materials and methods

### Animals

The present study utilized HIV-1 IIIB Tat_1–86_ transgenic female and male mice that were developed on a hybrid C57BL/6 J background (Chauhan et al., 2003; Bruce-Keller et al., 2008). HIV-Tat is brain-specific and expressed in astrocytes using a glial fibrillary acidic protein promoter under the control of a reverse tetracycline transactivator (*GFAP-rtTA*). Transgenic mice expressing the tetracycline response element (*TRE*)*-tat* gene were identified (Transnetyx, Inc., Cordova, TN) as transgenic Tat(+) mice and littermates lacking the *tat* transgenic gene were used as control Tat(−) mice. To induce Tat expression in Tat(+) mice, animals were fed an *ad libitum* doxycycline (DOX) chow (6 mg/g; product TD.09282, Envigo, NJ, United States) 1 month prior to and throughout the study duration. To control for off-target effects, the same DOX-chow was fed to control Tat(−) mice. Animals were kept in group housing, with 3–5 mice per cage, under a reversed 12-h light/dark cycle (with lights off at 6:00 a.m.) and had free access to water and chow.

Two cohorts of mice were used. First, behavioral and endocannabinoid experiments were conducted on a total of 38 Tat transgenic mice (20 female, 19 male; ~9–10 months of age). The second group consisted of 28 Tat transgenic mice (14 female, 14 male; ~5 months of age) and was used to confirm CBD and 7-hydroxy-CBD (CBD-7-COOH) concentration following an acute 30 mg/kg CBD injection.

All research procedures were conducted in strict accordance with the guidelines outlined in the NIH Guide for the Care and Use of Laboratory Animals (NIH Publication No. 85–23) and approved by the Institutional Animal Care and Use Committee (IACUC) at the University of North Carolina at Chapel Hill.

### Drugs

Cannabidiol (CBD; #90080, Cayman Chemical, Ann Arbor, MI) was dissolved in a vehicle solution composed of a mixture of ethanol, Kolliphor® EL (Sigma-Aldrich, #C5135, St. Louis, MO), and saline (0.9% NaCL, Cytiva, #Z1376, Marlborough, MA), in a 1:1:18 ratio. Administration of vehicle and CBD doses (3, 10, 30 mg/kg) was carried out through subcutaneous (s.c.) injections at a volume of 10 μL/g of body mass. These CBD doses were chosen as they have shown to improve cognition in rodents (Long et al., 2006, 2010) without adverse effects (Varvel et al., 2006). In all experiments, drug treatments were randomized.

### Experimental design

**Figure 1:**
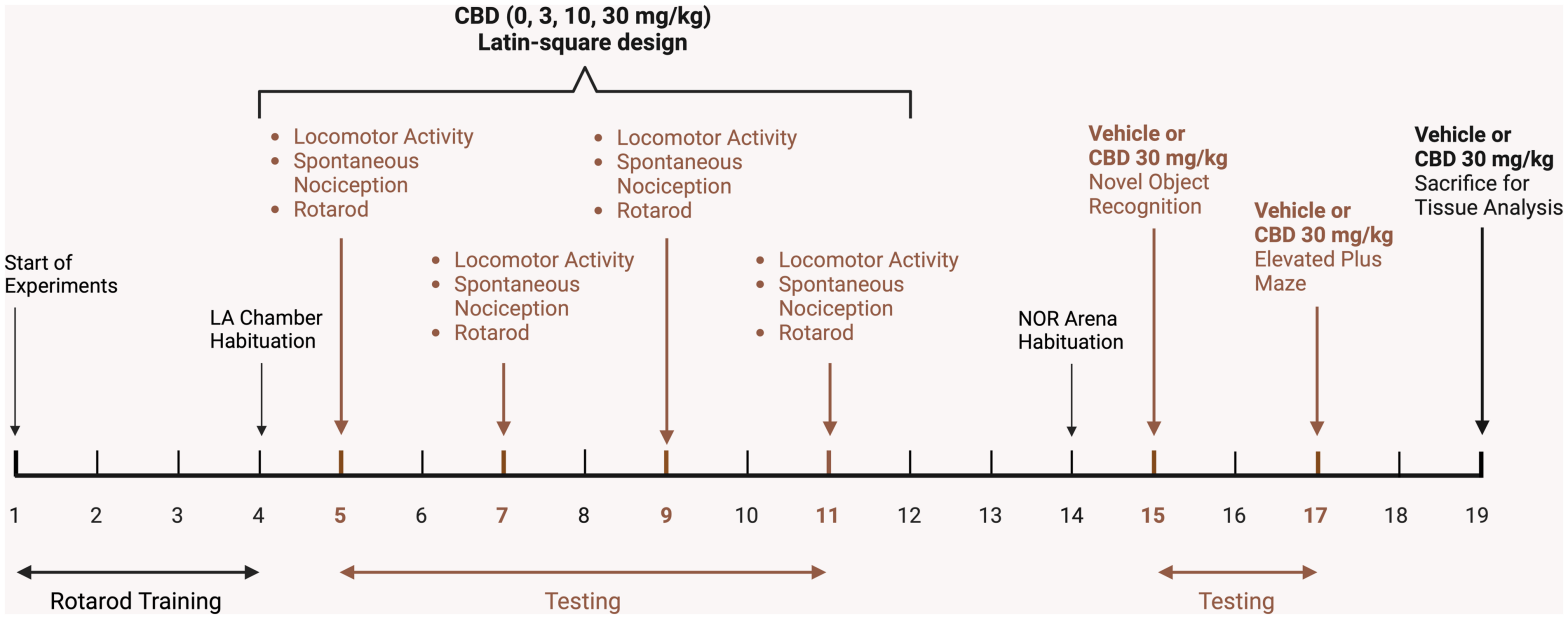
Schematic representation of the experimental study design on a timeline. After mice received DOX-containing chow for 1 month, mice were trained on the rotarod for 4 days and habituated to the locomotor activity chambers. Subsequently, vehicle and CBD doses (3, 10, 30 mg/kg) were administered in a Latin-square design over 4 days, with a minimum 48-h interval between testing days for CBD clearance. Locomotor activity (LA, 10 min) was conducted 60 min after injections, followed by assessment of spontaneous nociception, and lastly rotarod performance. Three days later mice were habituated (10 min) to the novel object recognition arena and trained and tested (each 10 min sessions) the following day in the novel object recognition (NOR) task, with vehicle or 30 mg/kg CBD injections being conducted after training but 60 min prior NOR testing. The elevated plus maze (EPM) task was conducted 2 days after NOR assessment with mice being injected with vehicle or 30 mg/kg CBD 60 min prior EPM testing (10 min). Two days later, mice were injected with vehicle or 30 mg/kg CBD and sacrificed 60 min post-injection for tissue analysis. LA, Locomotor activity; NOR, Novel object recognition. Figure created with BioRender.com.

The experimental design of the conducted study is outlined on a timeline in Figure 1. Mice underwent training in the rotarod task for 4 days (3 trials per day) and habituated to the testing chambers used for evaluating locomotor activity. Subsequently, CBD doses, including vehicle control (0, 3, 10, 30 mg/kg), were administered over the course of four consecutive days, following a Latin-square design. To ensure proper clearance of CBD, a minimum 48-h interval was maintained between each testing day. The assessment of locomotor activity, spontaneous nociception, and rotarod performance occurred 60, 75, and 90 min post-injection, respectively. Additionally, the novel object recognition (NOR) and elevated plus maze (EPM) tasks were conducted 4 and 6 days later, respectively, with either vehicle or 30 mg/kg CBD injections administered approximately 60 min prior to these tasks. Two days after behavioral experiments were completed, mice were injected with vehicle or 30 mg/kg CBD and sacrificed 60 min later for CNS tissue analysis. Drug injections and behavioral measures were typically carried out between 9 a.m. and 1 p.m. each day.

### Behavioral procedure

#### Spontaneous heat-evoked nociception

The tail-flick and hot-plate assays were employed to evaluate spontaneous heat-evoked nociception with a focus on spinal and supraspinal pathways, respectively (Singh et al., 2018).

For the tail-flick test, the distal 1/3 of each mouse’s tail was immersed in a water bath (56°C ± 0.1°C, Thermo Scientific, Precision General-Purpose Water Bath, Model 181, Waltham, MA). The latency to remove the tail from the bath was recorded as an indicator of nociception. To prevent tissue damage, the maximum cut-off time was 10 s.

The hot-plate test was conducted immediately following the tail-flick assay. Mice were placed on the surface of a hot plate (55 ± 0.1°C; IITC, Inc., MOD 39, Woodland Hills, CA) enclosed within a Plexiglas™ chamber (15 cm height, 10 cm diameter) to prevent escape. The mouse was removed from the hot plate as soon as it withdrew or licked a paw, or exhibited jumping behavior. The time spent on the hot plate was recorded as an indicator of nociception. To prevent tissue damage, the maximum cut-off time was 15 s.

#### Locomotor activity

The locomotor activity task was employed to evaluate spontaneous locomotion in mice. Mice were placed within standard mouse experimental chambers (MED Associates, ENV-307 W; 22 cm × 18 cm floor, Fairfax, VT), situated in sound and light attenuating compartments (MED Associates, ENV-022MD). Testing occurred in a darkened room, illuminated by red fluorescent lighting, with white noise from an air conditioning unit inside the room. The ambient temperature in the testing room was maintained at 22°C with a humidity level of 30%. Locomotor activity was recorded over a duration of 10 min (600 s) using night vision cameras (Amcrest, FullHD 1080P 2MP Dome, Houston, TX) installed on the ceiling of the soundproof chambers. Videos were recorded to a Security Recorder (Amcrest, AMDV8M16-H5). The chamber floor was divided into four equal sized and shaped quadrants, and the overall locomotor activity of mice was quantified by the number of times they moved their noses across the boundaries between these quadrants. Numerical data were generated from the 10 min video recordings by a team of trained experimenters blinded to treatment conditions. Each video was assessed by two experimenters independently, with an overall interrater reliability (Cronbach’s α) of α = 0.855. The locomotor activity data (# of crossings) represent the average score derived from the assessments of both experimenters.

#### Rotarod

The accelerating rotarod test was employed to evaluate motor function and coordination (Jones and Roberts, 1968). Mice were positioned on a rotarod apparatus (Harvard Apparatus, #76-0770, Holliston, MA) consisting of an elevated, rubber-coated rod (30 mm diameter, elevated 18 cm). The rod was divided into five sections (each 50 mm wide) allowing for testing of five mice simultaneously. Mice were placed on the rod and given 60 s to acclimate prior trial start. Subsequently, rods accelerated from 2 to 60 rpm over a period of 7 min (420 s). The duration (s) each animal stayed on the rotating rod without falling or looping was recorded.

#### Novel object recognition

The novel object recognition (NOR) task was employed to evaluate object recognition memory which relies on mice’s natural tendency to explore novel stimuli (Ennaceur, 2010; Lueptow, 2017). The task was conducted within a hexagonal arena constructed of lightly textured high-density white polyethylene (50 cm wide, 23 cm tall, courtesy of G.F. League Co., Inc., Greenville, SC). The task encompassed three phases: habituation, training, and testing, as previously detailed (Lueptow, 2017). Briefly, mice were individually habituated to the testing apparatus for 5 min and returned to their home cage. The training phase started 24–48 h later when mice were placed into the arena containing two identical objects (familiar objects) for 10 min before they were returned to their home cage. Two hours later, mice were injected (s.c.) with vehicle or CBD (30 mg/kg) and kept in their home cage for an additional 60 min before the testing phase started. For the testing phase, mice were placed again for 10 min in the same arena containing one familiar object and one novel object (randomized for each mouse). The duration of time that mice spent exploring the objects (familiar and novel) during the testing session was recorded with a video camera (GoPro Hero 6 Black, v02.10, San Mateo, CA) mounted overhead. Object exploration was defined as being in close proximity to the object and facing it at a distance closer than 1 cm. The glass objects utilized were equal in size (20 cm high, 7 cm wide) but differed in shape, with one being a square prism and the other a curved cylinder. The choice of object to serve as the familiar or novel one was randomized for each mouse. Numerical data were generated from the 10 min video recordings of the testing phase by a team of trained experimenters blinded to treatment conditions. Each video was assessed by two experimenters independently, with an overall interrater reliability of α = 0.949 for the novel object and α = 0.987 for the familiar object. For both dependent variables, data represent the average score derived from the assessments of both experimenters. Total exploration time (s) of both objects (familiar and novel) was calculated to determine object exploration time. A discrimination index (DI) was calculated according to the formula: DI = [t(novel) – t(familiar)]/[t(novel) + t(familiar)] for the testing phase (Lueptow, 2017; Miedel et al., 2017). A discrimination index of 0 indicates no preference, 1 indicates a complete preference for the novel object, and − 1 indicates a complete preference for the familiar object.

#### Elevated plus maze

The elevated plus maze (EPM) task was employed to evaluate anxiety-like behavior (Shoji and Miyakawa, 2021). The elevated, plus-shaped apparatus (San Diego Instruments, #7001–0316, San Diego, CA) consisted of two open arms and two closed arms (15 cm tall walls). Each arm was 30 cm long, 5 cm wide, and 38 cm above the ground. To ensure uniform illumination of all four arms, indirect lighting was employed. Mice were placed in the center of the apparatus and roaming activity was recorded for 10 min (600 s) with a video camera (GoPro, Hero 6 Black, v02.10, San Mateo, CA) mounted overhead. Numerical data were generated from the 10 min video recordings by a team of trained experimenters blinded to treatment conditions. The time spent in the open arms [presented as percent time spent in the open arms from total exploration time (600 s)] and number of nose pokes into the open arms served as indicators of anxiety-like behavior. Each video was assessed by two experimenters independently, with an overall interrater reliability of α = 0.976 for latency and α = 0.971 for number of pokes. For both dependent variables data represent the average score derived from the assessments of both experimenters.

### Analysis of endocannabinoids and related lipids

Two days upon completion of behavioral experiments, mice received an acute s.c. injection of either vehicle or CBD (30 mg/kg) and were sacrificed 60 min later by rapid decapitation following isoflurane-induced anesthesia. Four CNS regions from the right hemisphere (prefrontal cortex, striatum, cerebellum, and spinal cord) were dissected and snap-frozen in liquid nitrogen within 10 min of decapitation. Samples were stored at −80°C until use. Using ultraperformance liquid chromatography–tandem mass spectrometry (UPLC-MS/MS), the following lipids were quantified: the two main endocannabinoids *N*-arachidonoylethanolamine (AEA/anandamide) and 2-arachidonoylglycerol (2-AG), *two* related eicosanoids *N*-oleoyl ethanolamide (OEA) and *N*-palmitoyl ethanolamide (PEA), and arachidonic acid (AA). Details on the extraction and quantification of endocannabinoids and related lipids have been described previously (Dempsey et al., 2019; League et al., 2021) and are outlined in Supplementary Methods.

### Western blot analysis

Left hemisphere of Tat transgenic mice was used to quantify the expression levels of CB_1_R, CB_2_R, GPR55, FAAH, and MAGL. The brain tissue was homogenized in ice-cold Pierce™ RIPA lysis and extraction buffer (Thermo Scientific, Cat# 89900, United States) with Halt™ phosphatase (Thermo Scientific, Cat# 78420, United States) and protease inhibitor cocktail (Thermo Scientific, Cat# 87786, United States). Homogenized tissue lysates were centrifuged at 10,000 g for 10 min at 4°C. Pierce™ BCA protein assay kit (Thermo Scientific, Cat# 23227, United States) was used to determine the protein concentration. Following the protein quantification, protein lysates were suspended in sample buffer containing NuPAGE™ LDS Sample Buffer (Invitrogen™, Cat# NP0007, United States) and XT Reducing Agent (BioRad, Cat# 1610792, United States) in 1:2.5 ratio and denatured at 85°C for 10 min. Equal amounts of protein (20 μg/lane) were resolved in 10% Bis-Tris Criterion™ XT Precast Gels and XT MOPS running buffer (BioRad, Cat# 1610799, United States) at 120 volts for 1.5 h using Criterion™ vertical electrophoresis cell (BioRad, Cat# 1656001, United States). Electrophoretic transfer of proteins from the gel to nitrocellulose membranes (0.45 m BioRad, Cat#1620115, United States) was carried out in 10x Tris/Glycine buffer (BioRad, Cat# 1610734) at 100 volts for 1 h using Criterion™ blotter with wire electrodes (BioRad, Cat#1704071, United States) at 1°C–4°C. Blots were rinsed with phosphate-buffered saline (PBS) and incubated with Intercept® blocking buffer (LI-COR Biosciences, Cat# 727-70,001) at room temperature for 1 h. Blots were incubated with primary antibodies overnight at 4°C in Intercept® blocking buffer with 0.2% Tween-20. Primary antibodies used in this study were, anti-CB_1_R (rabbit polyclonal; Proteintech, Cat# 17978-1-AP, 1:1,000 dilution), anti-CB_2_R (rabbit polyclonal; ABclonal, Cat# A1762, 1:1,000 dilution), anti-GPR55 (rabbit polyclonal; ABclonal, Cat# A12890, 1:1,000 dilution) anti-FAAH (rabbit monoclonal; ABclonal, Cat# A4099, 1:1,000 dilution), and anti-MAGL (rabbit polyclonal; ABclonal, Cat# A23306, 1:1,000 dilution). Anti-GAPDH antibody (mouse monoclonal; Abcam, Cat# ab125247, 1:15,000 dilution) was used as a housekeeping protein. Next day, the blots were washed 3x with PBST (PBS with 0.1% Tween-20) followed by incubation with the secondary antibodies. The secondary antibodies used were, IRDye® 680RD Donkey anti-Mouse IgG (LI-COR Biosciences, Cat# 926-68,072, 1:15,000 dilution) and IRDye® 800CW Donkey anti-Rabbit IgG (LI-COR Biosciences, Cat# 925-32,213, 1:15,000 dilution) secondary antibodies at room temperature for 1 h in Intercept® blocking buffer with 0.2% Tween-20 and 0.01% SDS. Following the secondary antibody incubation, the blots were washed 3× with PBST and bands were detected using Odyssey® CLx infrared imaging system (LI-COR Biosciences, United States). Empiria studio® software version 2.3.0 (LI-COR Biosciences, United States) was used to analyze the images. Data represent the fold-change with respect to a control sample represented on all blots and normalized to the housekeeping gene GAPDH.

### Analysis of CBD and CBD-7-COOH concentration

A new cohort of drug-naive Tat transgenic female and male mice (*n* = 7 per sex) received an injection of an acute CBD dose (30 mg/kg, s.c.). This was done to confirm the concentration of CBD and its metabolite 7-hydroxy-CBD (CBD-7-COOH) in both plasma and mouse cortex. 60 min after CBD injections mice were sacrificed under isoflurane-induced anesthesia, and samples were collected within 5 min. Plasma samples were prepared from fresh drawn blood obtained via cardiac puncture (Supplementary Methods). After cardiac puncture, cortex samples were dissected and snap-frozen in liquid nitrogen immediately upon decapitation. Samples were stored at −80°C until further analysis.

Extraction and quantification of CBD and CBD-7-COOH concentration in plasma and cortex samples were carried out as follows. A 100 μL aliquot of plasma was extracted using 600 μL of a mixture of 80:20 MeOH:H_2_O. Samples were shaken for 15 min and then centrifuged for 10 min at 20,000 rcf. The resulting supernatant was dried down and subsequently reconstituted with 100 μL of MeOH. Cortex tissue was extracted using 600 μL of 80:20 MeOH:H_2_O, sonicated for 15 min to facilitate tissue breakdown, and then centrifuged for 10 min at 20,000 rcf. Cortex extracts were dried down and reconstituted in 100 μL MeOH.

The analysis of these samples was performed using a Waters ACQUITY Ultra-Performance Liquid Chromatography (UPLC) system tandem to a Thermo Scientific TSQ Vantage triple quadrupole mass spectrometer. Separations were achieved using a Waters BEH C18 150 mm x 2.1 mm column; water with 0.1% formic acid was used for mobile phase A and acetonitrile with 0.1% formic acid was mobile phase B. The flow rate was set at 0.25 mL/min, and the initial starting conditions were 65% A. A linear decrease was performed to 45% A over 2 min followed by a hold for 1 min. Another decrease to 20% A at 7 min was performed followed by another to 5% A at 8 min. A sharp decrease was performed from 5% to 0% A over 2 min with a curve of 3 (curve of 6 is linear). There was a hold at 100% B for 3 min followed by a re-equilibration step from 13.5 to 16 min. The injection volume was set at 10 μL. To monitor specific transitions for CBD and CBD-7-COOH, multiple reaction monitoring (MRM) was utilized (Supplementary Table S1).

### Statistical analysis

All data are presented as mean ± the standard error of the mean (SEM). Due to a lack of sex differences noted, datasets for spontaneous nociception, locomotor activity, and rotarod performance were collapsed across sex, and analyzed by two-way mixed analysis of variances (ANOVAs) with drug dose (4 levels: vehicle, 3, 10, 30 mg/kg CBD) as a within-subjects factor and genotype [2 levels: Tat(−) mice, Tat(+) mice] as a between-subjects factor. Datasets on novel object recognition, anxiety-related behavior, and Western blot analyses of cannabinoid receptors (CB_1_R, CB_2_R, GPR55) and endocannabinoid catabolic enzymes (MAGL, FAAH) were analyzed by three-way ANOVAs with drug (2 levels: vehicle, 30 mg/kg CBD), sex (2 levels: females, males), and genotype [2 levels: Tat(−) mice, Tat(+) mice] as between-subjects factors. Datasets for endocannabinoids and related lipid molecules were analyzed for each lipid molecule by one-way repeated ANOVAs with CNS regions [4 levels: prefrontal cortex, striatum, cerebellum, spinal cord] as a within-subjects factor. This was followed by a three-way multivariate analysis of variances (MANOVAs) conducted separately for each lipid molecule, with CNS regions as the multivariate variable and three between-subjects factors, including drug (2 levels: vehicle, 30 mg/kg CBD), sex (2 levels: females, males), and genotype [2 levels: Tat(−) mice, Tat(+) mice]. Datasets for plasma and cortex CBD concentration and its metabolites were analyzed by two-way ANOVAs with sex (2 levels: females, males) and genotype [2 levels: Tat(−) mice, Tat(+) mice] as between-subjects factors. For all ANOVAs, main or interaction effects, were followed by Tukey’s *post hoc* tests when appropriate. An alpha level of *p* ≤ 0.05 was considered significant for all statistical tests. SPSS Statistics 25 (IBM, Chicago, IL) and Prism GraphPad 8.0 (San Diego, CA) was used for data analysis and data graphing, respectively.

## Results

### Spontaneous heat-evoked nociception

**Figure 2:**
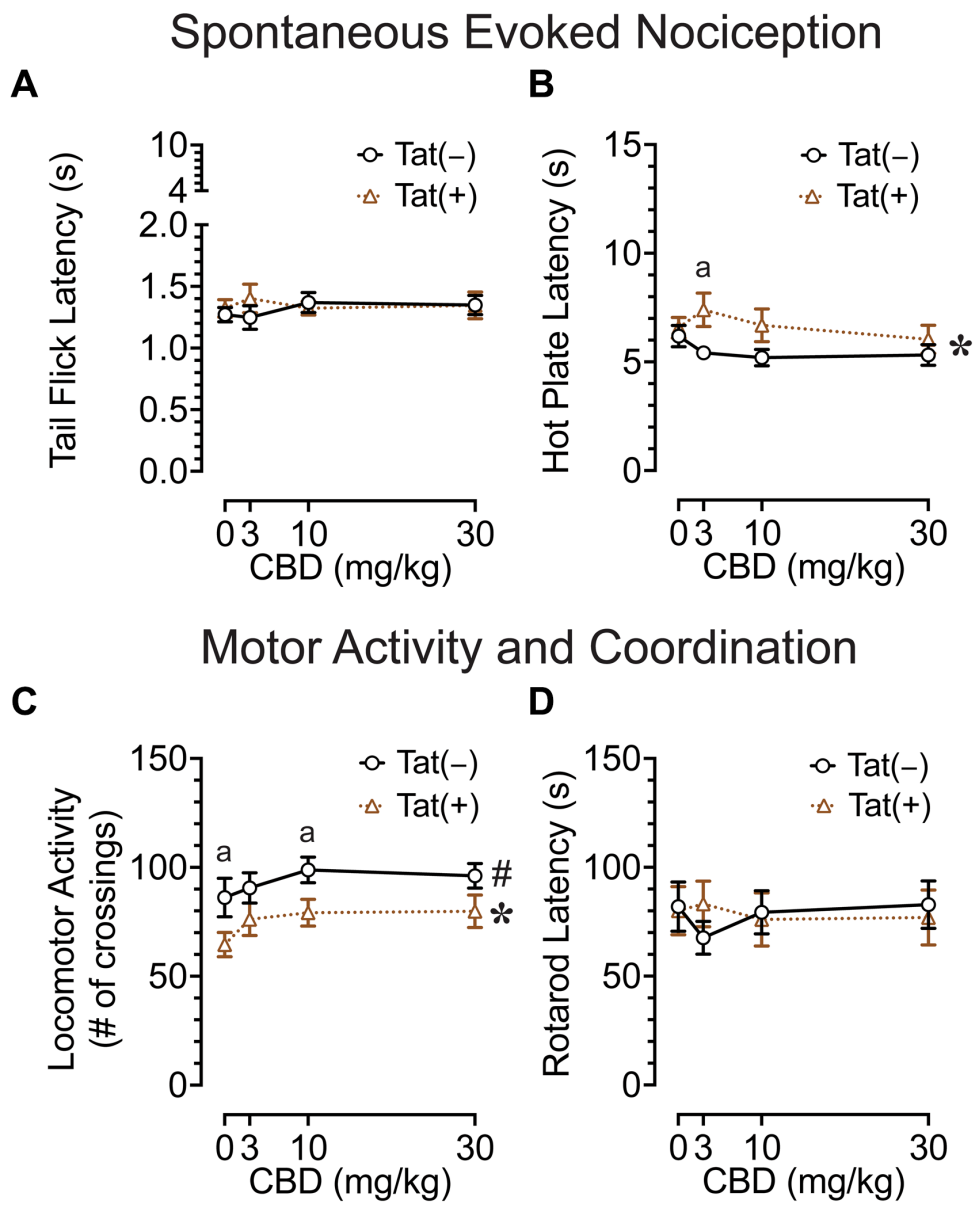
Effects of Tat expression and acute CBD doses (0, 3, 10, and 30 mg/kg) on spontaneous evoked nociception and motor function. **(A)** For the spinal-related tail-flick assay, no significant effects and/or interactions were noted. **(B)** For the supraspinal-related hot-plate assay, spontaneous nociception was altered by genotype, with group differences only being noted at 3 mg/kg CBD exposure, with higher latencies to pain signals for Tat(+) mice compared to Tat(−) mice. **(C)** Locomotor activity was altered by drug doses and genotype, but significant differences between groups were only noted for genotype, with decreased locomotor activity by Tat expression at vehicle and 10 mg/kg CBD exposure. **(D)** Motor coordination was not affected by Tat expression or acute CBD. All data are expressed as mean ± the standard error of the mean (SEM). Statistical significance was assessed by ANOVAs followed by Tukey’s *post hoc* tests when appropriate; *p < 0.05 main effect of genotype; ^#^*p* = 0.018 main effect of drug doses; ^a^*p* ≤ 0.05 Tat(+) mice vs. Tat(−) mice. *n* = 19–20 mice per group.

The tail-flick and hot-plate assays were conducted 75 min after drug injections to evaluate effects of acute CBD (0, 3, 10, and 30 mg/kg) exposure on heat-evoked pain-like behaviors in the Tat transgenic mouse model (*n* = 19–20 (10f) per group; Figures 2A,B). The tail-flick test was used to assess spinal-related spontaneous nociception (Figure 2A). A two-way mixed ANOVA demonstrated no significant effects or interactions.

The hot-plate test was used to assess supraspinal-related spontaneous nociception (Figure 2B). A two-way ANOVA demonstrated a significant main effect of genotype, *F*(1, 37) = 4.7, *p* = 0.039, with Tat(+) mice showing higher latencies to withdrawal or lick their paw (6.7 ± 0.46, *n* = 19) compared to Tat(−) mice (5.5 ± 0.29, *n* = 20), which was only significant for the 3 mg/kg CBD-exposed group as demonstrated by Tukey’s *post hoc* tests (*p* = 0.019). Follow-up Tukey’s *post hoc* comparisons for Tat(−) mice and Tat(+) mice revealed no statistical significant changes in spontaneous nociception when comparing vehicle to 3 mg/kg CBD injections. No other effects or interactions were significant.

Overall, spontaneous heat-evoked nociception was not affected by CBD doses. Interestingly, the demonstrated alteration in spontaneous nociception based on Tat expression in the supraspinal-related hot-plate test was only noted when animals received an acute 3 mg/kg CBD dose injection, as CBD had the non-significant tendency to decrease spontaneous nociception for Tat(+) mice, whereas the opposite effect was noted for Tat(−) mice.

### Locomotor activity and rotarod coordination

To understand the effects of acute CBD (0, 3, 10, and 30 mg/kg) exposure on motor function, we assessed locomotor activity and rotarod performance 60 and 90 min after injections, respectively [*n* = 19–20 (10f) per group; Figures 2C,D]. The locomotor activity task was conducted to assess effects of acute CBD in Tat transgenic mice on motor activity (Figure 2C). A two-way ANOVA demonstrated a significant main effect of drug doses, *F*(3, 111) = 3.5, *p* = 0.018, with increased locomotion for all three CBD doses compared to vehicle (Vehicle, 75.6 ± 5.53, *n* = 39; 3 mg/kg CBD, 83.5 ± 5.17, *n* = 39; 10 mg/kg CBD, 89.2 ± 4.48, *n* = 39; 30 mg/kg CBD, 88.2 ± 4.78, *n* = 39). Tukey’s *post hoc* tests demonstrated no significant differences between doses. Further, a significant main effect of genotype was noted, *F*(1, 37) = 5.3, *p* = 0.027, with Tat(+) mice showing lower locomotor activity compared to Tat(−) mice. Tukey’s *post hoc* tests demonstrated reduced locomotor activity for Tat(+) mice compared to Tat(−) mice when animals were exposed to vehicle (*p* = 0.050) and 10 mg/kg CBD (*p* = 0.026). No other effects or interactions were significant.

The rotarod task was conducted to investigate effects of acute CBD exposure in Tat transgenic mice on motor coordination and function (Figure 2D). A two-way mixed ANOVA demonstrated no significant effects or interactions.

Overall, an increase in locomotor activity was noted for CBD doses without affecting rotarod performance. Further, Tat expression decreased locomotor activity in mice when exposed to vehicle and 10 mg/kg CBD.

### Novel object recognition

**Figure 3:**
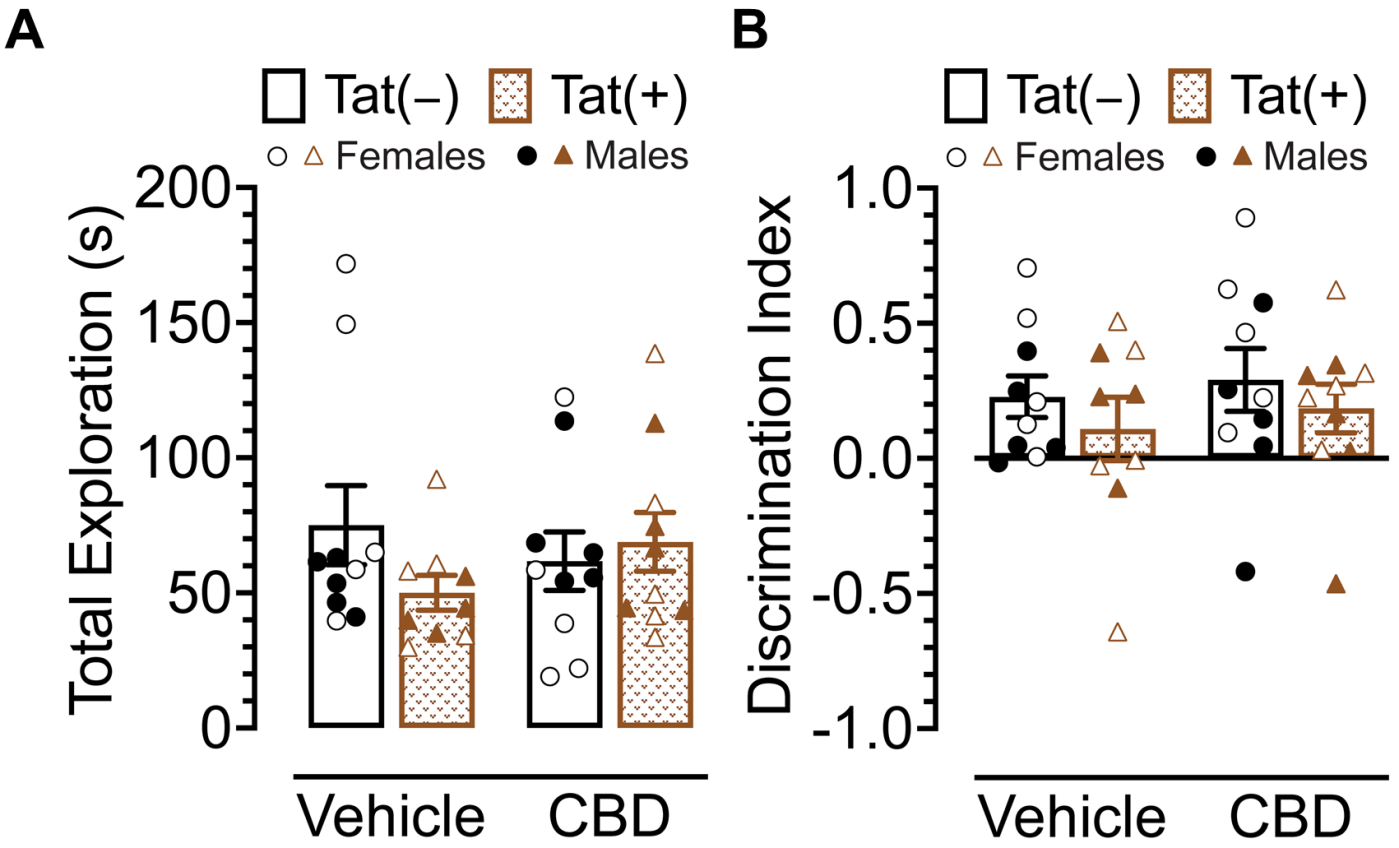
Novel object recognition memory was not altered by Tat expression or acute CBD (30 mg/kg) exposure. **(A)** Total object (novel and familiar objects) exploration time was similar across all groups. **(B)** Novel object recognition memory, indicated by the discrimination index (0 = no preference, 1 = complete preference for the novel object, −1 = complete preference for the familiar object) demonstrated no significant effects. All data are expressed as mean ± the standard error of the mean (SEM). Statistical significance was assessed by ANOVAs. CBD dose = 30 mg/kg. *n* = 9–10 mice per group; open circles and open triangles are female data points, closed circles and closed triangles are male data points.

The NOR task was conducted 60 min after injections to evaluate the effects of acute CBD (0 and 30 mg/kg) exposure in Tat transgenic mice on novel object recognition memory (*n* = 9–10 (5f) per group; Figure 3). The dependent measures included total exploration time of the novel and familiar objects and time spent exploring the novel object over the familiar object (discrimination index). For total exploration time (Figure 3A) a three-way ANOVA demonstrated no significant effects or interactions. Similarly, for object recognition memory, which is the discrimination index demonstrated by complete preference for the novel object equal to 1, no preference equal to 0, and complete preference for the familiar object equal to −1 (Figure 3B), a three-way ANOVA demonstrated no significant effects or interactions.

Overall, Tat expression and CBD appear to have no effects on novel object recognition memory.

### Anxiety-like behavior

**Figure 4:**
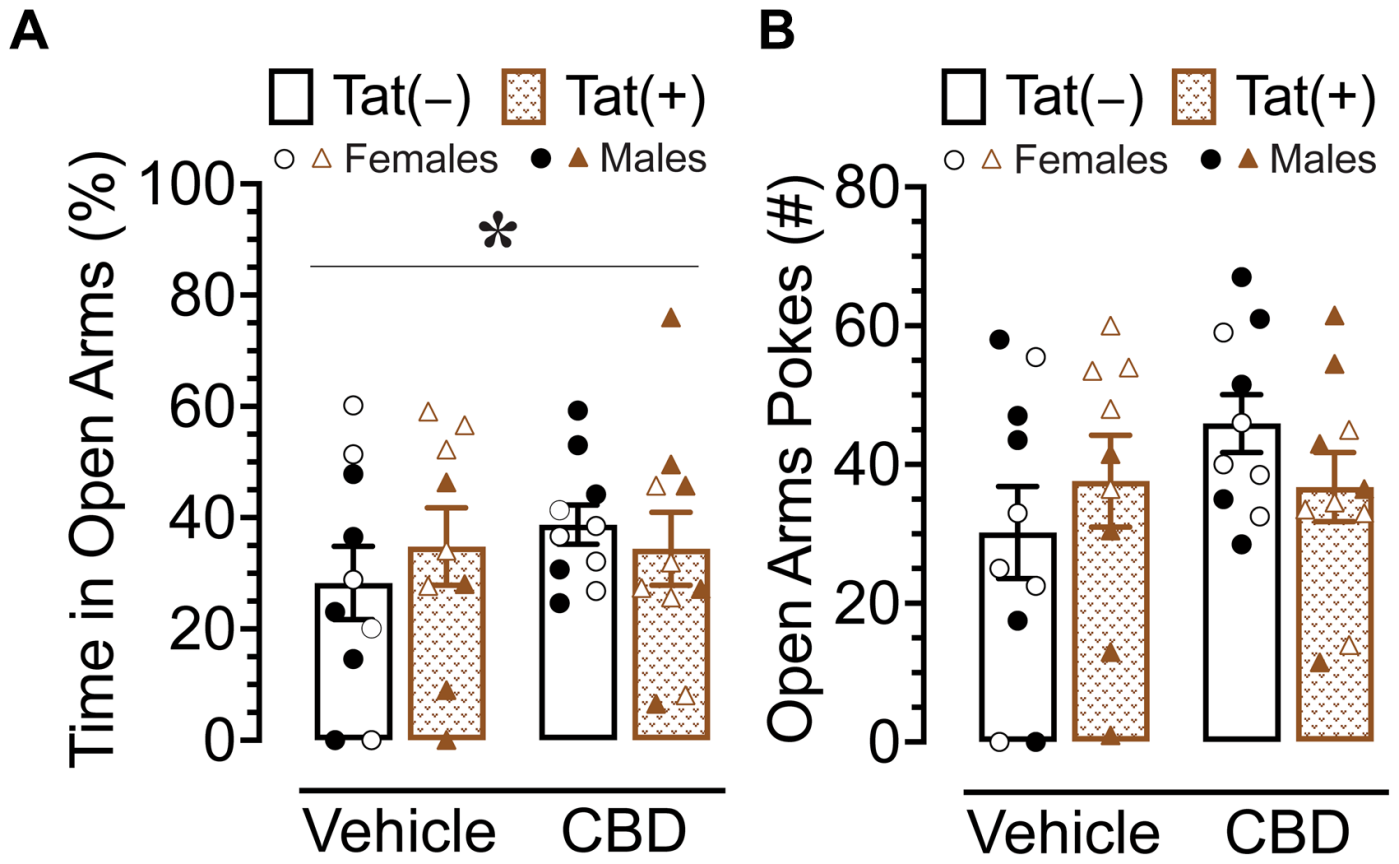
Anxiety-like behavior assessed by the elevated plus maze demonstrated differential effects of CBD (30 mg/kg) based on sex. **(A)** Percent time spent in open arms was altered by sex x drug, with CBD decreasing anxiety-like behavior in males only, but no significant group differences were noted for multiple comparisons. **(B)** No significant effects were noted for number of pokes into open arms. All data are expressed as mean ± the standard error of the mean (SEM). Statistical significance was assessed by ANOVAs followed by Tukey’s *post hoc* tests when appropriate. *p = 0.029 sex × drug interaction. CBD dose = 30 mg/kg. *n* = 9–10 mice per group; open circles and open triangles are female data points, closed circles and closed triangles are male data points.

The elevated plus maze task was conducted 60 min after injections to evaluate the effects of acute CBD (0 and 30 mg/kg) exposure in Tat transgenic mice on anxiety-like behavior (*n* = 9–10 (5f) per group; Figure 4). The dependent measures included percent time spent in open arms and number of pokes into open arms. For percent time spent in open arms (Figure 4A) a three-way ANOVA revealed a significant sex x drug interaction, *F*(1, 37) = 5.2, *p* = 0.029, in which 30 mg/kg CBD decreased anxiety-like behavior in males but not female mice. Tukey’s *post hoc* comparisons revealed no significant differences between any of the groups.

For number of pokes into open arms (Figure 4A), no effects or interactions were found to be significant.

Overall, CBD appears to have differential effects in male and female mice, with decreasing anxiety-like behavior in male mice but not in female mice.

### CNS levels of endocannabinoids and related lipids

**Figure 5:**
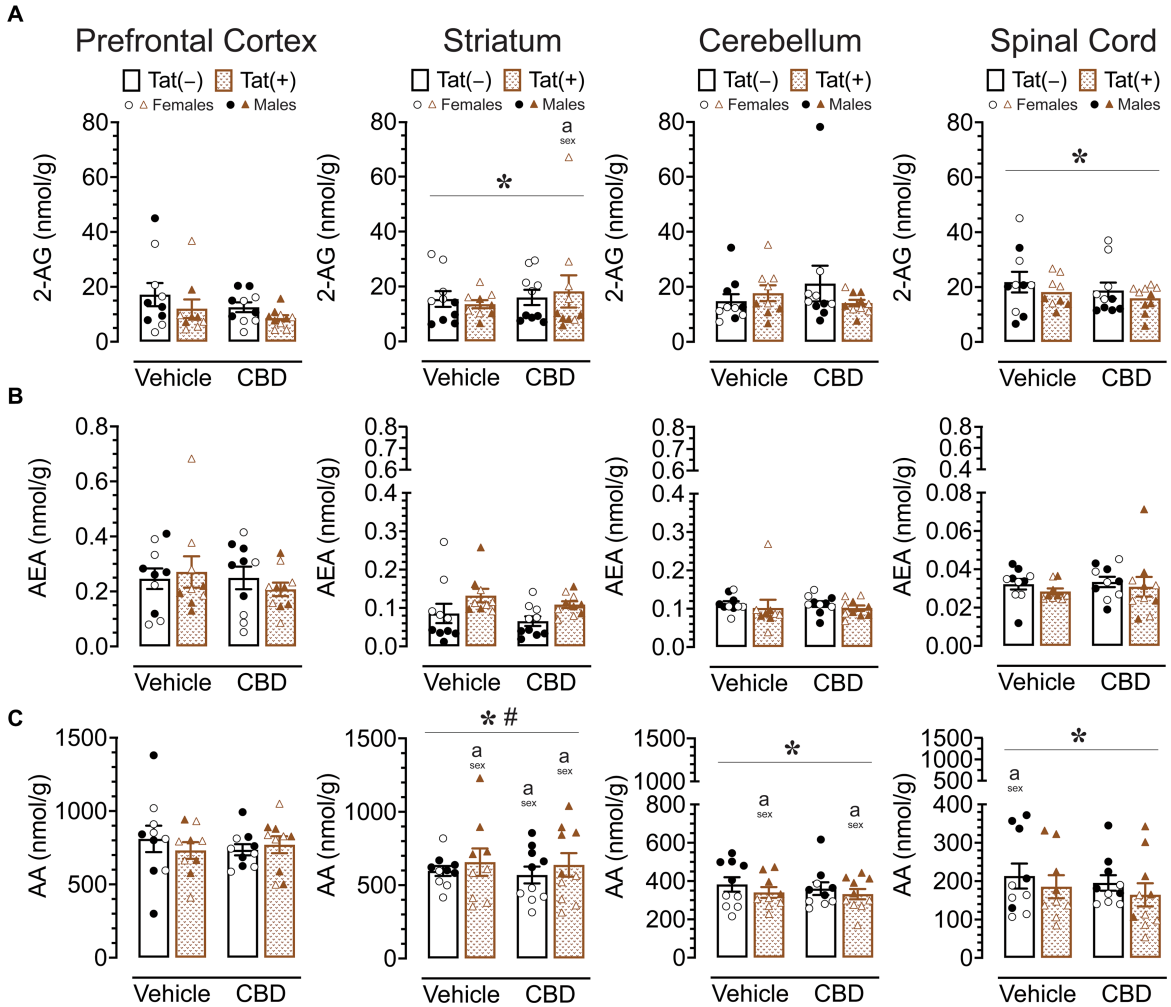
2-AG, AEA, and AA levels (nmol/g) were not affected by acute CBD (30 mg/kg) exposure and only minimal effects were noted for Tat expression on AA, but 2-AG and AA demonstrated sex differences in CNS regions. Concentration of 2-AG, AEA, and AA were assessed in the prefrontal cortex, striatum, cerebellum, and spinal cord for vehicle and acute CBD-treated Tat transgenic mice via LC/MS/MS. Lipid concentration were normalized to nmol/g of tissue. Note that no effects or interactions were noted for acute CBD administration on any measure. **(A)** 2-AG levels in the striatum and spinal cord were altered by sex, but significant differences between groups were only noted for the striatum, with higher 2-AG levels in CBD-exposed female mice compared to males. **(B)** AEA levels demonstrated no significant effects for any CNS region. **(C)** AA levels demonstrated a significant main effect of sex for the striatum, cerebellum, and spinal cord. AA levels in the striatum, cerebellum, and spinal cord were significantly lower in female mice compared to males in specific groups. Also, a significant sex x genotype interaction was noted for the striatum, but no significance group differences were found for multiple comparisons. All data are expressed as mean ± the standard error of the mean (SEM). Statistical significance was assessed by ANOVAs followed by Tukey’s *post hoc* tests when appropriate; *p < 0.05 main effect of sex; ^#^*p* = 0.009 sex × genotype interaction; ^a^*p* < 0.05 females vs. males within the group specified by the vertical bar. CBD dose = 30 mg/kg. *n* = 9–10 mice per group; open circles and open triangles are female data points, closed circles and closed triangles are male data points.

To assess the impact of acute CBD (0 and 30 mg/kg) exposure on the endogenous cannabinoid system, changes in levels of 2-AG, AEA, PEA, OEA, and AA were assessed 60 min after injections in various CNS regions of Tat transgenic mice (*n* = 9–10 (5f) per group), including the prefrontal cortex, striatum, cerebellum, and spinal cord (Supplementary Table S2; Figure 5). One-way repeated ANOVAs for each lipid molecule demonstrated lipid molecule concentration (nmol/g) differed significantly between CNS region for AEA, PEA, OEA, and AA, but not for 2-AG. AEA, *F*(3, 114) = 66.0, *p* < 0.001, demonstrated differences in expression levels between all CNS regions (*p*’s < 0.001), except between the striatum and cerebellum, with highest AEA levels found in the prefrontal cortex, followed by the striatum and cerebellum, and the lowest AEA levels being expressed in the spinal cord. The same differences were seen for PEA, *F*(3, 114) = 162.9, *p* < 0.001, and OEA, *F*(3, 114) = 172.2, *p* < 0.001. AA, *F*(3, 114) = 122.2, *p* < 0.001, also showed differences in expression levels between all CNS regions (*p*’s < 0.001), except between the prefrontal cortex and striatum, with highest AA levels found in the prefrontal cortex and striatum, followed by the cerebellum, and the lowest AA levels being expressed in the spinal cord (Supplementary Table S2; Figure 5). To assess treatment effects, a MANOVA was conducted for each lipid molecule with drug, sex, and genotype as between-subjects factors. No effects or interactions were noted for acute CBD administration on any measure, indicating that acute CBD did not alter the endocannabinoid system and related lipid molecules. The most prominent findings were noted for AA levels with some minor effects for 2-AG (Figure 5), PEA and OEA (Supplementary Table S2), and no effects for AEA (Figure 5).

For 2-AG (Figure 5A), significant main effects of sex were noted for the striatum, *F* (1, 31) = 18.5, *p* < 0.001, and spinal cord, *F*(1, 31) = 12.7, *p* = 0.001, with females demonstrating higher 2-AG levels compared to male mice in both CNS regions. No other significant effects or interactions were noted. Tukey’s *post hoc* tests for each CNS sample demonstrated only a significant difference in the striatum between CBD-exposed Tat(+) females compared to CBD-exposed Tat(+) males, with females demonstrating higher 2-AG levels compared to male mice (*p* = 0.045).

For AEA (Figure 5B), no significant effects or interactions were noted.

For AA (Figure 5C), a significant sex effect was noted for the striatum, *F*(1, 31) = 50.8, *p* < 0.001, with males demonstrating higher AA levels compared to female mice. Further, a significant sex x genotype interaction was noted, *F*(1, 31) = 7.8, *p* = 0.009, with Tat(+) male mice showing higher AA levels in the striatum compared to Tat(−) males (*p* = 0.020), which was not noted for females. Tukey’s *post hoc* tests demonstrated higher AA levels in males compared to female mice, for vehicle-exposed Tat(+) mice (*p* < 0.001), CBD-exposed Tat(−) mice (*p* = 0.013), and CBD-exposed Tat(+) mice (*p* < 0.001). No significant differences were noted across drug or genotype within each sex. Further, a significant sex effect was noted for the cerebellum, *F*(1, 31) = 56.1, *p* < 0.001, and the spinal cord, *F*(1, 31) = 31.5, *p* < 0.001, with females demonstrating lower AA levels compared to male mice for both CNS regions. Tukey’s *post hoc* tests demonstrated lower AA levels in females compared to male mice, in the cerebellum, for vehicle-exposed Tat(+) mice (*p* = 0.050), and CBD-exposed Tat(+) mice (*p* = 0.027), and in the spinal cord for vehicle-exposed Tat(−) mice (*p* = 0.054).

Results for PEA and OEA mirrored a few of the findings for AA, specifically the noted main effects of sex in the striatum [PEA, *F*(1,31) = 20.0, *p* < 0.001; OEA, *F*(1, 31) = 15.6, *p* < 0.001] and cerebellum [PEA, *F*(1, 31) = 9.7, *p* = 0.004; OEA, *F*(1, 31) = 5.7, *p* = 0.023], with lower PEA and OEA levels for females compared to males; additionally a main effect of genotype was demonstrated in the spinal cord for PEA, *F*(1, 31) = 8.0, *p* = 0.008, and OEA, *F*(1, 31) = 11.4, *p* = 0.002, with lower levels for Tat(+) mice compared to Tat(−) mice (Supplementary Table S2).

Overall, acute CBD administration did not alter endocannabinoids or related lipid molecules in any CNS region. The most prominent effects were noted for AA expression levels with males showing higher AA levels compared to females in all CNS regions except the prefrontal cortex. Whereas no significant effects were noted for AEA, 2-AG levels were higher for females compared to male mice in the striatum and spinal cord.

### Expression levels of cannabinoid receptors and endocannabinoid degradative enzymes

#### CB_1_R, CB_2_R, GPR55, MAGL, and FAAH protein expression levels in the striatum

**Figure 6:**
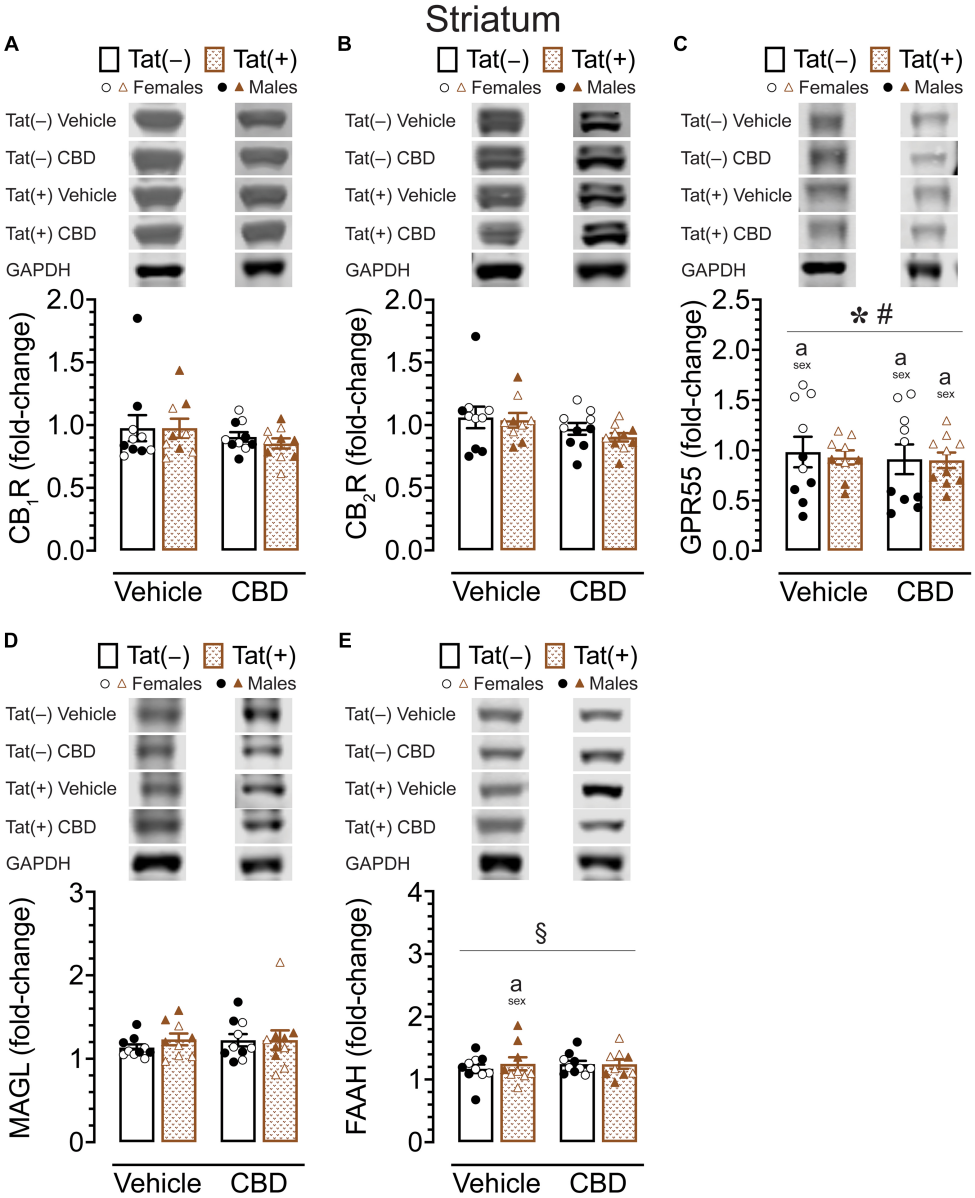
CB_1_R, CB_2_R, GPR55, MAGL and FAAH expression levels in the striatum. CB_1_R, CB_2_R, GPR55, MAGL, and FAAH expression levels were assessed in the striatum of vehicle and acute CBD-exposed Tat transgenic mice via Western blot. Data were normalized to the housekeeping protein GAPDH and fold-change was calculated using a control sample represented on all blots. **(A)** Representative immunoblots for CB_1_R and GAPDH (top). CB_1_R protein expression was not significantly altered in any group (bar graph, bottom). **(B)** Representative immunoblots for CB_2_R and GAPDH (top). CB_2_R expression was not significantly altered in any group (bar graph, bottom). **(C)** Representative immunoblots for GPR55 and GAPDH (top). GPR55 protein expression was significantly altered by sex and sex x genotype, but significant differences between groups were only noted for sex, with upregulated GPR55 expression levels in female mice compared to males for vehicle-exposed Tat(−) mice, CBD-exposed Tat(−) mice, and CBD-exposed Tat(+) mice (bar graph, bottom). **(D)** Representative immunoblots for MAGL and GAPDH (top). MAGL expression was not significantly altered in any group (bar graph, bottom). **(E)** Representative immunoblots for FAAH and GAPDH (top). FAAH expression demonstrated a significant drug x sex x genotype interaction, with group differences only been noted for vehicle-exposed Tat(+) mice, with females demonstrating less FAAH expression compared to male mice (bar graph, bottom). All data are expressed as mean ± the standard error of the mean (SEM). Statistical significance was assessed by ANOVAs followed by Tukey’s *post hoc* comparisons when appropriate; *p < 0.001 main effect of sex; ^#^*p* = 0.001 sex × genotype interaction; ^§^*p* = 0.005 drug × sex × genotype interaction; ^a^*p* < 0.05 females vs. males within the group specified by the vertical bar. CBD dose = 30 mg/kg. *n* = 9–10 mice per group; open circles and open triangles are female data points, closed circles and closed triangles are male data points.

To assess the impact of acute CBD (0 and 30 mg/kg) exposure on cannabinoid receptors and cannabinoid catabolic enzymes, changes in protein expression levels of CB_1_R, CB_2_R, GPR55, MAGL, and FAAH were assessed 60 min after injections in the striatum of Tat transgenic mice (*n* = 9–10 (5f) per group; Figure 6). Original and unedited Western blot gels of protein expression levels of CB_1_R, CB_2_R, GPR55, MAGL, and FAAH are shown in Supplementary Figures S1–S3. Data were normalized to the housekeeping protein GAPDH and fold-change was calculated using a control sample represented on all blots. For CB_1_R protein expression (Figure 6A) and CB_2_R protein expression (Figure 6B), a three-way ANOVA demonstrated no significant main or interaction effects.

For GPR55 protein expression (Figure 6C), a three-way ANOVA demonstrated a significant main effect of sex, *F*(1, 31) = 91.95, *p* < 0.001, with females demonstrating higher GPR55 expression levels compared to male mice. Interestingly, the sex effect was altered by genotype, significant sex x genotype interaction: *F*(1, 31) = 12.71, *p* = 0.001. Tukey’s *post hoc* tests demonstrated higher GPR55 expression levels in females compared to male mice, for vehicle-exposed Tat(−) mice (*p* < 0.001), for CBD-exposed Tat(−) mice (*p* < 0.001), and for CBD-exposed Tat(+) mice (*p* = 0.023).

For MAGL enzyme expression (Figure 6D) a three-way ANOVA demonstrated no significant main or interaction effects.

For FAAH enzyme expression (Figure 6E), a three-way ANOVA demonstrated a significant drug x sex x genotype interaction, *F*(1, 31) = 9.37, *p* = 0.005. Tukey’s *post hoc* tests demonstrated lower FAAH enzyme expression in vehicle-exposed Tat(+) females compared to vehicle-exposed Tat(+) males (*p* = 0.045).

Overall, whereas no or minimal effects were noted for CB_1_R, CB_2_R, MAGL, and FAAH expression in the striatum, significant higher GPR55 expression levels were noted in females compared to male mice within most of the individual drug and genotype groups.

#### CB_1_R, CB_2_R, GPR55, MAGL, and FAAH protein expression levels in the cerebellum

**Figure 7:**
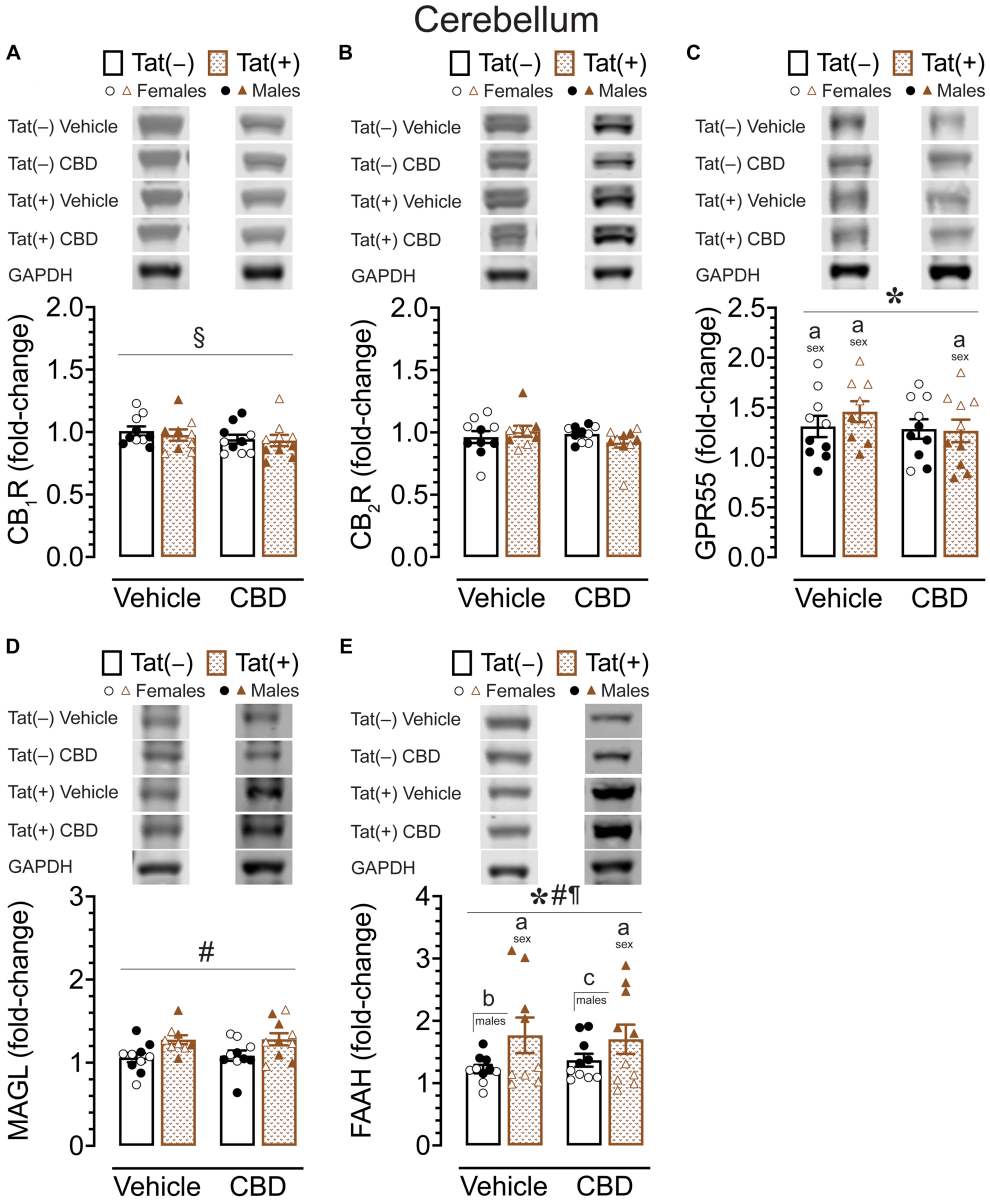
CB_1_R, CB_2_R, GPR55, MAGL and FAAH expression levels in the cerebellum. CB_1_R, CB_2_R, GPR55, MAGL, and FAAH expression levels were assessed in the cerebellum of vehicle and acute CBD-treated Tat transgenic mice via Western blot. Data were normalized to the housekeeping protein GAPDH and fold-change was calculated using a control sample represented on all blots. **(A)** Representative immunoblots for CB_1_R and GAPDH (top). CB_1_R protein expression demonstrated a significant drug x sex x genotype interaction, but no significant group differences were noted for multiple comparisons (bar graph, bottom). **(B)** Representative immunoblots for CB_2_R and GAPDH (top). CB_2_R expression was not significantly altered in any group (bar graph, bottom). **(C)** Representative immunoblots for GPR55 and GAPDH (top). GPR55 expression was significantly altered by sex, with upregulated GPR55 expression levels in female mice compared to males for vehicle-exposed Tat(−) mice, vehicle-exposed Tat(+) mice, and CBD-exposed Tat(+) mice (bar graph, bottom). **(D)** Representative immunoblots for MAGL and GAPDH (top). MAGL expression demonstrated a significant genotype effect, but no significant group differences were noted for multiple comparisons (bar graph, bottom). **(E)** Representative immunoblots for FAAH and GAPDH (top). FAAH expression demonstrated a significant effect of genotype, sex, and genotype x sex interaction, with lower FAAH expression levels in female mice compared to males for vehicle-exposed Tat(+) mice, and CBD-exposed Tat(+) mice (bar graph, bottom). Further, higher FAAH enzyme expression levels were noted in vehicle-exposed Tat(+) males compared to vehicle-exposed Tat(−) males, and in CBD-exposed Tat(+) males compared to CBD-exposed Tat(−) males (bar graph, bottom). All data are expressed as mean ± the standard error of the mean (SEM). Statistical significance was assessed by ANOVAs followed by Tukey’s hoc comparisons when appropriate; *p < 0.001 main effect of sex; ^#^*p* < 0.01 main effect of genotype; ^¶^*p* < 0.001 sex x genotype interaction; ^§^*p* < 0.001 drug x sex x genotype interaction; ^a^*p* < 0.05 females vs. males within the group specified by the vertical bar; ^b^*p* < 0.001 vehicle-exposed Tat(−) males compared to vehicle-exposed Tat(+) males; ^c^*p* = 0.009 in CBD-exposed Tat(−) males compared to CBD-exposed Tat(+) males. CBD dose = 30 mg/kg. *n* = 9–10 mice per group; open circles and open triangles are female data points, closed circles and closed triangles are male data points.

To assess the impact of acute CBD (0 and 30 mg/kg) exposure on cannabinoid receptors and cannabinoid catabolic enzymes, changes in protein expression levels of CB_1_R, CB_2_R, GPR55, MAGL, and FAAH were assessed 60 min after injections in the cerebellum of Tat transgenic female and male mice (*n* = 9–10 (5f) per group; Figure 7). Original and unedited Western blot gels of protein expression levels of CB_1_R, CB_2_R, GPR55, MAGL, and FAAH are shown in Supplementary Figures S4–S6. Data were normalized to the housekeeping protein GAPDH and fold-change was calculated using a control sample represented on all blots. For CB_1_R protein expression (Figure 7A), a three-way ANOVA demonstrated a significant drug x sex x genotype interaction, *F*(1, 31) = 17.34, *p* < 0.001. Tukey’s *post hoc* tests demonstrated no significant differences between any groups.

For CB_2_R protein expression (Figure 7B), a three-way ANOVA demonstrated no significant main or interaction effects.

For GPR55 protein expression (Figure 7C), a three-way ANOVA demonstrated a significant main effect of sex, *F*(1, 31) = 49.49, *p* < 0.001, with females demonstrating higher GPR55 expression levels compared to male mice. Tukey’s *post hoc* tests demonstrated higher GPR55 expression levels in females compared to male mice, for vehicle-exposed Tat(−) mice (*p* = 0.008), for vehicle-exposed Tat(+) mice (*p* = 0.045), and for CBD-exposed Tat(+) mice (*p* = 0.002).

For MAGL enzyme expression (Figure 7D) a three-way ANOVA demonstrated a significant main effect of genotype, *F*(1, 31) = 11.09, *p* = 0.002. Tukey’s *post hoc* tests demonstrated no significant differences between any groups.

For FAAH enzyme expression (Figure 7E), a three-way ANOVA demonstrated a significant main effect of sex, *F*(1, 31) = 91.21, *p* < 0.001, a significant main effect of genotype, *F*(1, 31) = 27.40, *p* < 0.001, and a significant sex × genotype interaction, *F*(1, 31) = 28.99, *p* < 0.001. Tukey’s *post hoc* tests demonstrated lower FAAH enzyme expression levels in females compared to male mice, for vehicle-exposed Tat(+) mice (*p* < 0.001), and for CBD-exposed Tat(+) mice (*p* < 0.001). Further, higher FAAH enzyme expression levels were noted in vehicle-exposed Tat(+) males compared to vehicle-exposed Tat(−) males (*p* < 0.001), and in CBD-exposed Tat(+) males compared to CBD-exposed Tat(−) males (*p* = 0.009).

Overall, similar to the striatum, the cerebellum also demonstrated significant higher GPR55 expression levels in females compared to male mice, with no or minimal effects for CB_1_R, CB_2_R, and MAGL expression. However, for FAAH expression male Tat(+) mice demonstrated higher FAAH enzyme expression levels compared to their Tat(+) female counterparts as well as compared to Tat(−) males.

#### Concentration of CBD and its metabolite CBD-7-COOH in plasma and cortex

**Figure 8:**
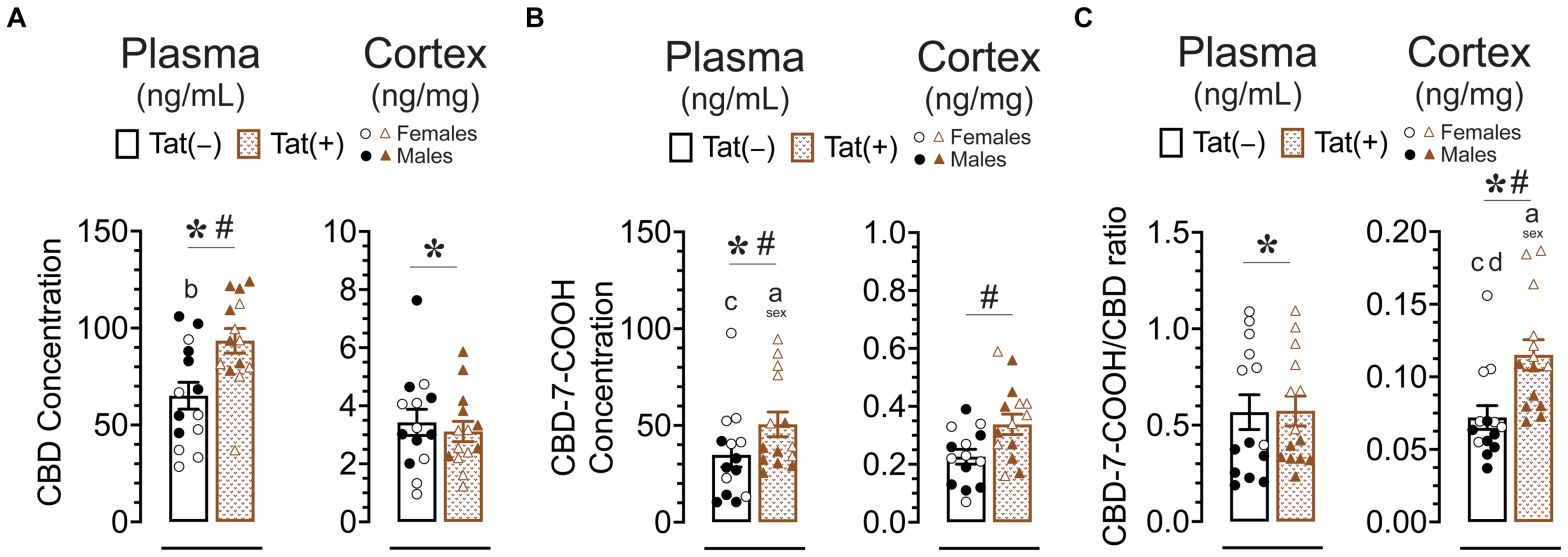
CBD, the CBD metabolite CBD-7-COOH concentration, and the CBD-7-COOH/CBD ratio after acute CBD (30 mg/kg) exposure are altered by genotype and/or sex. Concentration of CBD and CBD-7-COOH, and CBD-7-COOH/CBD ratios were assessed 60 min after 30 mg/kg CBD administration. Concentration are expressed as ng/mL for plasma and ng/mg for cortex samples. Concentration and ratios for all measures were higher in the plasma compared to the cortex. **(A)** CBD concentration in the plasma were significantly altered by sex and genotype, with group comparisons indicating higher plasma CBD concentration for Tat(+) males compared to Tat(−) female mice. CBD concentration in the cortex were significantly altered by sex, with lower cortex CBD concentration in females compared to males. **(B)** CBD-7-COOH concentration in the plasma were significantly altered by sex and genotype, with group comparisons indicating higher plasma CBD-7-COOH concentration for Tat(+) females compared to Tat(+) male and Tat(−) male mice. CBD-7-COOH concentration in the cortex were significantly altered by genotype, with higher CBD-7-COOH concentration in Tat(+) mice compared to Tat(−) mice. **(C)** CBD-7-COOH/CBD ratio in the plasma was significantly altered by sex, with higher CBD-7-COOH/CBD ratio in females compared to males. CBD-7-COOH/CBD ratio in the cortex was significantly altered by sex and genotype, with group comparisons indicating higher CBD-7-COOH/CBD ratios in Tat(+) females compared to all other groups. All data are expressed as mean ± the standard error of the mean (SEM). Statistical significance was assessed by ANOVAs followed by Tukey’s *post hoc* tests when appropriate; *p < 0.05 main effect of sex; ^#^*p* < 0.05 main effect of genotype; ^a^*p* ≤ 0.05 females vs. males within the group specified by the vertical bar; ^b^*p* = 0.001 Tat(−) females vs. Tat(+) males; ^c^*p* < 0.01 Tat(−) males compared to Tat(+) female; ^d^*p* = 0.004 Tat(−) females vs. Tat(+) females; CBD dose = 30 mg/kg. *n* = 14 mice per group; open circles and open triangles are female data points, closed circles and closed triangles are male data points.

Concentration of CBD and CBD-7-COOH were assessed 60 min after acute CBD (30 mg/kg) exposure using a different cohort of animals. Tat transgenic mice (*n* = 14 (7f) per group) were subcutaneously injected with 30 mg/kg CBD and sacrificed 60 min later, around the time when behavior was assessed (Figure 8). CBD concentration and its metabolite CBD-7-COOH were detected in all plasma (ng/mL) and cortex (ng/mg) samples. The CBD-7-COOH/CBD ratio was further calculated for all plasma and cortex samples to get a better understanding of metabolite concentration. A two-way ANOVA was conducted with sex and genotype as between-subjects factors for plasma and cortex samples separately.

For CBD concentration (Figure 8A) a significant main effect of genotype was noted in plasma samples, *F*(1, 21) = 11.25, *p* = 0.003, with higher plasma CBD concentration in Tat(+) mice compared to Tat(−) mice. Further, a significant main effect of sex, *F*(1, 24) = 8.01, *p* = 0.009, demonstrated lower plasma CBD concentration in females compared to males. Tukey’s *post hoc* tests revealed significant higher plasma CBD concentration for Tat(+) males compared to Tat(−) female mice (*p* = 0.001). For cortex samples, CBD concentration demonstrated a significant main effect of sex, *F*(1, 24) = 5.62, *p* = 0.026, with lower cortex CBD concentration in females compared to males.

For CBD-7-COOH concentration (Figure 8B), a significant main effect of genotype was noted in plasma samples, *F*(1, 24) = 4.48, *p* = 0.045, with higher plasma CBD-7-COOH concentration in Tat(+) mice compared to Tat(−) mice. Further, a significant main effect of sex was noted, *F*(1, 24) = 13.00, *p* = 0.001, demonstrating higher plasma CBD-7-COOH concentration in females compared to males. Tukey’s *post hoc* tests revealed higher plasma CBD-7-COOH concentration in Tat(+) females compared to Tat(+) males (*p* = 0.031) and Tat(+) females compared to Tat(−) males (*p* = 0.002). For cortex CBD-7-COOH concentration a significant main effect of genotype was noted, *F*(1, 24) = 6.04, *p* = 0.022, with higher cortex CBD-7-COOH concentration in Tat(+) mice compared to Tat(−) mice.

For plasma CBD-7-COOH/CBD ratio (Figure 8C) a significant main effect of sex was noted, *F*(1, 24) = 67.96, *p* < 0.001, with higher plasma CBD-7-COOH/CBD ratio in female mice compared to males. For cortex samples, CBD-7-COOH/CBD ratio revealed a significant main effect of genotype, *F*(1, 24) = 18.87, *p* < 0.001, with higher cortex CBD-7-COOH/CBD ratio in Tat(+) mice compared to Tat(−) mice. Further, a significant main effect of sex, *F*(1, 24) = 21.36, *p* < 0.021, demonstrated higher cortex CBD-7-COOH/CBD ratio in females compared to males. Tukey’s *post hoc* tests revealed higher cortex CBD-7-COOH/CBD ratio in Tat(+) females compared to all other groups, including Tat(−) females (*p* = 0.004), Tat(+) males (*p* = 0.002), and Tat(−) males (*p* < 0.001).

Overall, Tat expression increased plasma CBD, plasma CBD-7-COOH, and cortex CBD-7-COOH concentration. Additionally, whereas CBD-7-COOH/CBD ratio did not differ in plasma samples, cortex samples showed a higher CBD-7-COOH/CBD ratio in Tat(+) mice compared to Tat(−) mice, which is in favor of an increase in metabolite concentration in the cortex. Further, sex differences were noted for CBD-7-COOH/CBD ratios, in which females compared to males displayed higher plasma and cortex CBD-7-COOH/CBD ratios in Tat(+) and/or Tat(−) mouse groups.

## Discussion

The present study investigated acute effects of CBD in Tat transgenic female and male mice on various behavioral measures and the endocannabinoid system. HIV Tat induction decreased locomotor activity and lowered pain sensitivity without affecting levels of endocannabinoids and related lipids. Acute CBD overall produced little effects on behavior, only altering locomotor activity and anxiety-like behavior, and had no effects on the endocannabinoid system. Sex effects were noted for the endocannabinoid system and related lipids, including elevated 2-AG levels for females in the striatum, and upregulated AA levels for males in the striatum, cerebellum, and spinal cord. Further, sex effects were noted for GPR55 being expressed at higher levels in females compared to males in the striatum and cerebellum, whereas FAAH expression levels were higher in Tat(+) males compared to Tat(+) females. Even though Tat expression did not alter acute CBD effects on any behavioral task and endocannabinoid measure, CBD metabolism was significantly affected by sex and Tat induction.

Chronic pain is commonly reported by PLWH (Lu et al., 2021; Slawek, 2021) and significantly interferes with daily function (Namisango et al., 2012). Preclinical studies using the Tat transgenic mouse model have reported mechanical allodynia, thermal hyperalgesia, and damage to nerve fibers due to Tat expression (Chi et al., 2011; Wodarski et al., 2018; Cirino et al., 2021), but others have found either no effects (Fitting et al., 2012) or decreased pain sensitivity (Bagdas et al., 2020). The current study supports the finding that Tat induction attenuates pain sensitivity but only at the supraspinal level without affecting the spinal-related tail flick task. Based on the variable reports, it is clear that Tat’s effects on nociception are complex and highly dose- and time-dependent (Bagdas et al., 2020; Toma et al., 2022), as well as assay-dependent (i.e., mechanical, thermal, or cold pain sensitivity; Toma et al., 2022). A previous study reported hyposensitivity to mechanical pain in Tat(+) mice following Tat induction for 3 weeks (Bagdas et al., 2020), similar to our 1 month Tat induction paradigm. However, hypersensitivity to mechanical pain was reported in transgenic mice when Tat was expressed for more than 1 month (Wodarski et al., 2018; Toma et al., 2022). It has been suggested that Tat’s neurotoxic effects on nociceptive neurons (Chi et al., 2011; Wodarski et al., 2018) potentially induce nerve dysfunction during an initial period, resulting in diminished pain signaling, however, this later reverses into pain hypersensitivity due to prolonged nerve damage (Bagdas et al., 2020). The finding of reduced pain sensitivity for the supraspinal-related hot plate test but not the spinal-related tail flick test may be explained by a previous study that found higher Tat mRNA expression in the striatum compared to the spinal cord (Fitting et al., 2012). For acute CBD exposure, no effects on pain sensitivity in Tat transgenic mice were found at any dose given (3, 10, 30 mg/kg), even though other studies demonstrated attenuation of pain sensitivity by acute CBD treatment of similar doses in preclinical rodent models of inflammatory pain (Philpott et al., 2017; Wanasuntronwong et al., 2022) and neuropathic pain (Xiong et al., 2012; Jesus et al., 2019). To our knowledge, cannabidiol’s effects on pain have not been studied in PLWH and/or neuroHIV mouse models, including the Tat transgenic mouse model. However, it has been reported that cannabis use in PLWH improves nerve pain (Woolridge et al., 2005), shrinks the area of painfully sensitive skin (Abrams et al., 2007), and can relief neuropathic pain (Ellis et al., 2009). Further, CBD has been shown to be necessary for THC-related antinociception in some human trials (Johnson et al., 2010). Thus, to provide more insights into possible mechanisms underlying CBD’s effects on pain sensitivity, future studies should focus on chronic CBD use as well as investigate the implications of various CBD:THC ratios among PLWH and in preclinical neuroHIV mouse models, including the Tat transgenic mice.

Besides chronic pain as a common comorbidity among PLWH, individuals also exhibit motor deficits, including impaired gait, multitasking, motor strength, and motor coordination, all correlated with HAND status (Kronemer et al., 2017; Robinson-Papp et al., 2020). As a result of these motor difficulties, physical activity often suffers, and a majority of PLWH exhibit inadequate levels of daily physical activity, even lower than most other chronic disease populations (Vancampfort et al., 2018). These lower levels of activity have been associated with depression, physical pain, and worse cardio-respiratory fitness (Vancampfort et al., 2018; Ceccarelli et al., 2020). In accordance with this, preclinical neuroHIV rodent models, including Tat transgenic mice, have been found to exhibit lower levels of locomotor activity (June et al., 2009; Moran et al., 2013; Joshi et al., 2020). This finding is supported by the current study that demonstrated lower locomotor activity for Tat(+) mice compared to control Tat(−) mice, despite intact motor coordination. For CBD, the present study found that CBD doses increased locomotor activity independent of Tat expression, even though group comparisons did not confirm this finding, and rotarod performance was also not affected by CBD. Multiple preclinical studies have reported the lack of CBD effects on locomotor activity in non-diseased mice (Viudez-Martinez et al., 2019; Poulia et al., 2021) and disease rodent models (Zieba et al., 2019; Coles et al., 2020; Florensa-Zanuy et al., 2021), including motor performance in the rotarod test (Schleicher et al., 2019; Viudez-Martinez et al., 2019), which is supported by a recent review on effects of CBD on locomotor activity (Calapai et al., 2022). Even though we did not see differential effects of CBD based on Tat expression, a previous study reported reduction of hypolocomotion by chronic treatment of CBD via 5-HT1A receptors, and thus improvement in motor dysfunction caused by hepatic encephalopathy (Magen et al., 2010), which suggests CBD can increase locomotor activity. Nevertheless, further research is necessary to better understand CBD’s effects on locomotion in the context of neuroHIV, especially in the light of rodent models that include multiple HIV proteins and not just a single viral protein, such as Tat.

Another noteworthy aspect of CBD is its known anxiolytic effects (Blessing et al., 2015; Bonaccorso et al., 2019; Zieba et al., 2019; Garcia-Baos et al., 2021; Huffstetler et al., 2023), which were partially present in the current study as differential CBD effects were noted for sex, with decreasing anxiety-like behavior in male mice but not in female mice, even though follow-up group comparisons revealed no significance. Sex differences in the effectiveness of CBD as a potential treatment for anxiety in a clinical setting still have to be explored (Wright et al., 2020), but preclinical studies have shown that anxiolytic effects can be achieved by acute CBD administration in both sexes (Fabris et al., 2022; Huffstetler et al., 2023). However, the effectiveness of CBD appears to be sex-dependent as previous research has shown that the CBD dose required to produce a decrease in anxiety-like behavior was lower in females compared to male mice (Huffstetler et al., 2023). Interestingly, a recent study demonstrated that the responsiveness of female rats to acute CBD depended on the stage of the estrous cycle, with female rats being responsive only in the late diestrus phase at a 10-fold lower dose than males, but females were unresponsive to acute CBD in the proestrus phase (Fabris et al., 2022). Further, a bell-shaped dose–response relationship is usually reported for CBD effects on anxiety-like behavior (Salviato et al., 2021; Fabris et al., 2022), with anxiolytic effects of CBD being observed at lower doses (10 mg/kg) but less or no effects at higher doses (Guimaraes et al., 1990; Onaivi et al., 1990). In the future, it would be interesting to monitor estrous cycle in female mice as well as use a range of CBD doses to get a better understanding of CBD’s effects on anxiety-like behavior in Tat transgenic mice.

An unexpected finding was the lack of Tat and CBD effects on the memory-related object recognition memory task. Previous research has shown that tests of learning and memory typically show memory deficits in mice exposed to Tat (Carey et al., 2012; Marks et al., 2021), and CBD administration has been shown to attenuate disease-associated memory deficits in a variety of contexts (Fagherazzi et al., 2012; Zhornitsky and Potvin, 2012; Cheng et al., 2014; Osborne et al., 2017a,b; Stark et al., 2019; Razavi et al., 2020; Garcia-Baos et al., 2021). The lack of CBD effects may be due to the fact that Tat(+) mice in the current study did not show deficits in the object recognition task. Another caveat was that control mice did not successfully differentiate the novel object from the familiar object, which was noted specifically in Tat(−) male mice. This may be due to similarity between the two objects used. Follow-up investigation could use objects that differ significantly in odor, size, color/brightness, or texture to ensure that mice can differentiate the two.

For the endocannabinoid system, the current study found no acute CBD or Tat effects. A caveat of the present study was that animals were not drug naive when they received the final acute injection prior to tissue harvest (see Figure 1), which may need to be kept in mind when interpreting the present findings on the endocannabinoid system. Nevertheless, previous preclinical studies have reported the lack of acute CBD exposure at different doses to affect brain endocannabinoids and related lipids [despite high CBD concentration in blood and whole brain (Mustafa et al., 2023)]. In contrast, multiple studies demonstrated changes in endocannabinoid levels and related lipids following Tat exposure (Hermes et al., 2018, 2021; Xu et al., 2022) as well as Tat-induced changes in CB_1_R, CB_2_R, and FAAH enzyme expression (Jacobs et al., 2019; Yadav-Samudrala et al., 2023). Alterations of the endocannabinoid system in postmortem tissue of PLWH has been demonstrated previously with reported changes in CB_1_R and CB_2_R expression in the frontal lobe (Cosenza-Nashat et al., 2011; Swinton et al., 2021), as well as increases in FAAH expression in cortical postmortem tissue samples from rhesus monkeys infected with simian immunodeficiency virus displaying encephalitis (Benito et al., 2005). The upregulation of AA levels in the striatum and cerebellum of Tat(+) male mice is not surprising as AA plays an important role in pro- and anti-inflammatory responses (Bosetti, 2007; Tallima and El Ridi, 2018; Wang et al., 2021), and chronic Tat and HIV-1 infection in the CNS increases inflammatory processes via upregulation of inflammatory mediators and glia activation (Hauser et al., 2007; Chen et al., 2017; Ajasin and Eugenin, 2020). Interestingly, the current study found sex-specific effects for the endocannabinoid system. For endocannabinoids and related lipids, male mice demonstrated higher AA levels compared to females in all CNS regions except the prefrontal cortex, and females compared to males demonstrated higher 2-AG levels in the striatum and partially in the spinal cord. GPR55 protein expression in the striatum and cerebellum was higher for females compared to males for most of the drug and genotype groups. Further, FAAH expression levels in the cerebellum were higher for Tat(+) males compared to Tat(+) females and also compared to Tat(−) males, which supports the finding of Tat(+) males displaying high AA levels in the cerebellum, potentially due to increased breakdown of AEA, which however demonstrated no differences in AEA expression based on sex or Tat expression in our study. More research is needed to understand how sex alters the endocannabinoid system in PLWH and how it relates to neuroinflammation and HAND, but vulnerabilities to HAND symptoms (Maki and Martin-Thormeyer, 2009; Maki et al., 2018; Sundermann et al., 2018; Rubin et al., 2019; Duarte et al., 2021) and immune activation (Ziegler and Altfeld, 2016; Santinelli et al., 2020) have been reported to be higher in women compared to men. Whereas a recent study did not find sex differences in various metabolites of the AA cascade of gp120 transgenic mice, transcriptomic analysis revealed sexual dimorphism of AA pathway-related genes, and females appear to be more responsive to the lipoxygenase pathway compared to males (Yuan et al., 2022). Of note is the current finding that females demonstrated overall higher 2-AG and GPR55 expression levels in the striatum, spinal cord, and/or cerebellum that in combination with the upregulated AA and FAAH expression levels in male mice of the similar CNS regions point to potential sex-dependent alterations in endocannabinoid degradation, which has been shown previously (Levine et al., 2021).

Even though we did not find differential effects of CBD based on Tat expression, CBD metabolite analyses demonstrated that CBD and CBD-7-COOH were differently metabolized by Tat(−) and Tat(+) mice and/or sex. The higher CBD/CBD-7-COOH ratios in plasma and cortex for females compared to males, suggests an increase in metabolite concentration for females. Two recent clinical studies that either administered CBD for 7 days or cannabis acutely did not find concentration differences for CBD and/or CBD-7-COOH in plasma of healthy individuals (Arkell et al., 2022; MacNair et al., 2023). However, the 7-day oral CBD administration study showed higher accumulation of CBD metabolites in plasma of females over time, which also is supported in a 12-week clinical CBD study (Batinic et al., 2023), suggesting sex differences in CBD metabolism or elimination (MacNair et al., 2023). As our study focused on acute CBD exposure (i.e., samples taken 60 min after CBD s.c. injections), it is suggested that faster metabolism in the plasma and brain may contributed to higher CBD/CBD-7-COOH ratios in females. In contrast, not much is known about how CBD is metabolized in PLWH or neuroHIV mouse models. The higher drug concentration in Tat(+) females and males, found in plasma for both CBD and CBD-7-COOH, and for cortex CBD-7-COOH might indicate overall alteration in CBD pharmacokinetics. Interestingly, when looking at plasma CBD/CBD-7-COOH ratio no differences were found for Tat status, which may indicate that Tat(−) mice store CBD faster but metabolism of CBD to CBD-7-COOH is the same in both genotypes due to the similar CBD/CBD-7-COOH ratios displayed. In fact, as CBD disperses more readily in a lipophilic environment, it should be pointed out that lipid abnormalities in PLWH have been reported previously (Funderburg and Mehta, 2016; Koethe et al., 2020) and adipose tissue is affected in HIV infection (Koethe, 2017). In contrast, in the cortex, higher cortex CBD/CBD-7-COOH ratios were found in Tat(+) mice compared to Tat(−) mice, which is in favor of an increase in metabolite concentration and could be interpreted as faster metabolism of CBD to CBD-7-COOH in the cortex or faster absorption to the brain, even though this is not shown for cortex CBD concentration. Future studies are necessary to investigate this in more detail, but overall, it suggests that HIV Tat status influences CBD pharmacokinetics, which if true, needs to be taken into account when considering CBD as a potential therapeutic treatment for PLWH.

## Data availability statement

The raw data supporting the conclusions of this article will be made available by the authors, without undue reservation.

## Ethics statement

The animal study was approved by Institutional Animal Care and Use Committee (IACUC) at the University of North Carolina at Chapel Hill. The study was conducted in accordance with the local legislation and institutional requirements.

## Author contributions

BY-S: Conceptualization, Data curation, Formal analysis, Investigation, Methodology, Project administration, Supervision, Validation, Visualization, Writing – original draft, Writing – review & editing. BG: Data curation, Formal analysis, Investigation, Project administration, Validation, Visualization, Writing – original draft, Writing – review & editing. KB: Data curation, Investigation, Validation, Writing – review & editing. HR: Data curation, Formal analysis, Investigation, Methodology, Validation, Visualization, Writing – original draft, Writing – review & editing. CH: Data curation, Investigation, Writing – review & editing. EW: Data curation, Writing – review & editing. MP: Data curation, Investigation, Writing – review & editing. JP: Data curation, Investigation, Writing – review & editing. WJ: Conceptualization, Data curation, Formal analysis, Funding acquisition, Methodology, Project administration, Resources, Supervision, Writing – review & editing. SF: Conceptualization, Data curation, Formal analysis, Funding acquisition, Investigation, Methodology, Project administration, Resources, Supervision, Validation, Visualization, Writing – original draft, Writing – review & editing.
