# Erratum: Effects of acute cannabidiol on behavior and the endocannabinoid system in HIV-1 Tat transgenic female and male mice

**DOI:** 10.3389/fnins.2025.1593198

**Published:** 2025-04-15

**Authors:** Frontiers Production Office

**Affiliations:** Frontiers Media SA, Lausanne, Switzerland

**Keywords:** cannabidiol, Tat transgenic mice, antinociception, 2-arachidonoylglycerol, arachidonic acid, GPR55, FAAH

Due to a production error, several references added to the article during production were not cited in the correct positions, resulting in the subsequent positions of reference citations throughout the article being incorrect. All reference citations from the **Introduction**, paragraph 3 onwards have been updated accordingly.

The publisher apologizes for this mistake. The original article has been updated.

