# Supplementary material for: Effects of acute cannabidiol on behavior and the endocannabinoid system in HIV-1 Tat transgenic female and male mice
Source: Front Neurosci. 2024 Mar 5;18:1358555. doi: 10.3389/fnins.2024.1358555 (PMC10949733; doi:10.3389/fnins.2024.1358555)
Supplement: Supplementary file 1 [file Data_Sheet_1.ZIP › AcuteCBD_Supplemental_Frontiers-REVISION.docx]

Supplementary Material

Effects of Acute Cannabidiol on Behavior and the Endocannabinoid System in HIV-1 Tat Transgenic Female and Male Mice

Barkha J. Yadav-Samudrala^1,*^, Benjamin L. Gorman^1^, Karenna M. Barmada^1^, Havilah P. Ravula^1^, Caitlin J. Huguely^1^, Diane Wallace^2^, Michelle R. Peace^3^, Justin L. Poklis^3^, Wei Jiang^4,5^, and Sylvia Fitting^1^

^1^Department of Psychology & Neuroscience, University of North Carolina at Chapel Hill, Chapel Hill, NC, 27599, USA; barkhaj@email.unc.edu (B.J.Y.-S.); bgorman@hms.harvard.edu (B.L.J.); kbarmada@unc.edu (K.M.B); hpr@unc.edu (H.P.R.); chuguely206@gmail.com (C.J.H.)

^2^Department of Chemistry, University of North Carolina at Chapel Hill, Chapel Hill, NC, 27599, USA; edianewallace@gmail.com (D.W.)

^3^Department of Pharmacology & Toxicology, Virginia Commonwealth University, Richmond, VA, 23298, USA; mrpeace@vcu.edu (M.R.P)

^4^Department of Microbiology and Immunology, Medical University of South Carolina, Charleston, SC, 29425, USA; jianw@musc.edu (W.J.)

^5^Division of Infectious Diseases, Department of Medicine, Medical University of South Carolina, Charleston, SC, 29425, USA; jianw@musc.edu (W.J.)

*****Correspondence: barkhaj@email.unc.edu; Tel.: +1-646-236-1901

Supplemental Material

1 Materials and Methods

*Analysis of endocannabinoids and related lipids*

2-AG, AEA, OEA, PEA, and AA were quantified via ultraperformance liquid chromatography-tandem mass spectrometry (UPLC-MS/MS) in four CNS regions, including the prefrontal cortex, striatum, cerebellum, and spinal cord, of male and female Tat transgenic mice, including the prefrontal cortex, striatum, cerebellum, and spinal cord (all samples were derived from the right hemisphere). Mice were sacrificed 90 min after drug injection (vehicle or 30 mg/kg CBD) via rapid decapitation following isoflurane-induced anesthesia. The four CNS regions were dissected, collected, and snap-frozen in liquid nitrogen within 10 min after decapitation. Samples were stored at −80 °C until the day of analysis. Samples from the right hemisphere were processed and substrates quantified in a similar manner to previous studies [1, 2]. Briefly, we prepared seven-point calibration curves encompassing the following concentrations for each lipid: 0.028 pmol to 2.8 pmol for AEA, 26 pmol to 2600 pmol for 2-AG, 3.1 pmol to 310 pmol for OEA, 3.3 to 330 pmol for PEA, and 0.33 nmol to 33 nmol for AA along with negative and blank controls. An additional internal standard (ISTD) was added to each calibrator, control, and sample except the blank control at concentrations of 0.28 pmol AEA-d8, 26 pmol 2-AG-d8, 31 OEA-d8, 33 PEA-d8, and 0.33 nmol AA-d8. The calibrator, control, and samples were analyzed as previously described [1]. Briefly, samples were homogenized in 100 μL ethanol and 900 μL water was then added. Sample cleanup was performed using UCT Clean Up® C18 solid phase extraction column (United Chemical Technologies, Inc., Bristol, PA) conditioned with methanol followed by water. Samples were added and the columns were then washed with deionized water and hexane. Lipids were eluted with 78:20:2 dichloromethane:isopropanol:ammonium hydroxide followed by ethyl acetate. The samples were then evaporated under nitrogen and reconstituted in mobile phase. A Sciex ExionLC system attached to a Sciex 6500 QTRAP system with an IonDrive Turbo V source for TurbolonSpray® (Sciex, Ontario, Canada) controlled by Analyst software (Sciex, Ontario, Canada) was used for the analysis of AEA, 2-AG, OEA, PEA, and AA.

Chromatographic separation of AEA, 2-AG, OEA, PEA, and AA was performed on a Discovery® HS C18 Column 15 cm × 2.1 mm, 3 μm (25 °C, Supelco: Bellefonte, PA). The mass spectrometer was operated in multiple reaction monitoring (MRM) positive ionization mode for AEA, 2-AG, OEA, PEA, and negative ionization mode for AA. The specific transition ions (m/z) and their corresponding collection energies (eV) were measured as follows: AEA: 348>62 (13) & 348>91 (60); AEA-d8: 356>63 (13); 2-AG: 379>287 (26) & 379>296 (28); 2-AG-d8: 384>287 (26); OEA: 326>62 (40) & 326 > 283 (40); OEA-d4 330 > 66 (40); PEA: 300 > 62 (31) & 300 > 283 (31); PEA-d4: 304 > 62 (31); AA: 303>259 (-25) & 303>59 (-60); AA-d8: 311>267 (-25). The total run time for the analytical method was 14 min. Calibration curves were analyzed within each analytical batch for each analyte. Linear regression of the ratio of the peak area counts for the analyte and corresponding deuterated ISTD vs. concentration was used to construct calibration curves.

*Plasma Sample Preparation*

Following isoflurane-induced anesthesia, 300 µL of blood was drawn from mice via cardiac puncture. Mice were sacrificed immediately after blood collection and the cortex was dissected for UPLC-MS/MS analysis. Blood was collected in 1.5 mL tubes kept on ice that contained 100 µL of EDTA dissolved in PBS (1x). EDTA dissolved in PBS (1x) was collected from EDTA-coated tubes (#366643, BD, Franklin Lakes, NJ, USA) that were rinsed with 500 µL cold PBS (1x). For plasma isolation, blood samples in 1.5 mL tubes were centrifuged at 1350 rcf at 4 °C for 20 min. The super layer of plasma was aspirated (100 µL) and stored at −80 °C until the day of analysis.

2 Supplemental Results

*Levels of PEA and OEA in the CNS*

To assess the impact of acute CBD (0 and 30 mg/kg) exposure on the endogenous cannabinoid system, changes in levels of PEA and OEA were assessed in various CNS regions of Tat transgenic female and male mice (n = 4–5 per group), including the prefrontal cortex, striatum, cerebellum, and spinal cord (Supplemental Table S2). A three-way multivariate ANOVA was conducted for each lipid molecule with drug, sex, and genotype as between-subjects factors. No effects or interactions were noted for acute CBD administration on any measure and are thus not shown. F-values and p-values for sex, genotype, and sex x genotype interaction are presented in Supplemental Table S2.

For PEA and OEA, significant sex effects were noted for the striatum and cerebellum with females demonstrating lower PEA and OEA levels compared to male mice. Further, significant genotype effects were noted for the spinal cord, with Tat(+) mice showing lower PEA and OEA levels compared to Tat(−) mice.

3 **Supplemental Tables and Figures**

**Table S1.** Multiple reaction monitoring transitions for standards. Quantifying transitions were used for quantitation, qualifying transitions were used to ensure proper identity.

| **Standard** | **Q1 *m/z*** | **Q3 *m/z*** | **Collision Energy (V)** | **Role** |
| --- | --- | --- | --- | --- |
| CBD | 315.2 | 193.1 | 22 | Quantifying |
| CBD | 315.2 | 123.0 | 32 | Qualifying |
| COOH-CBD | 345.2 | 327.2 | 15 | Quantifying |
| COOH-CBD | 345.2 | 299.2 | 19 | Qualifying |

**Table S2.** Effects of sex and genotype on PEA and OEA levels (nmol/g) in four CNS regions. Acute CBD administration (0 and 30 mg/kg) did not display any effects or interactions for any of the four CNS regions ^a^.

| **Lipids** | **CNS**  **Region** | **Sex** | **Geno-type** | **Vehicle** | **THC** | **Sex Effect** | | **Genotype Effect** | | **Sex x Genotype** | |
| --- | --- | --- | --- | --- | --- | --- | --- | --- | --- | --- | --- |
| **nmol/g** |  |  |  | **mean ± SEM** | **mean ± SEM** | ***F_1, 31_*** | ***p*** | ***F_1, 31_*** | ***p*** | ***F_1, 31_*** | ***p*** |
| **PEA** | **PFC** | Female | Tat(−) | 7.53 ± 0.82 | 6.40 ± 0.19 | 0.58 | 0.450 | 0.00 | 0.976 | 0.65 | 0.425 |
|  |  |  | Tat(+) | 6.97 ± 0.54 | 7.81 ± 0.74 |  |  |  |  |  |  |
|  |  | Male | Tat(−) | 7.58 ± 1.22 | 8.06 ± 0.74 |  |  |  |  |  |  |
|  |  |  | Tat(+) | 6.81 ± 0.84 | 7.92 ± 0.65 |  |  |  |  |  |  |
|  | **Str** | Female | Tat(−) | 3.76 ± 0.17 | 3.75 ± 0.18 | **20.04** | **<0.001** | 3.75 | 0.062 | 2.85 | 0.101 |
|  |  |  | Tat(+) | 3.59 ± 0.21 | 4.01 ± 0.22 |  |  |  |  |  |  |
|  |  | Male | Tat(−) | 4.28 ± 0.14 | 4.25 ± 0.16 |  |  |  |  |  |  |
|  |  |  | Tat(+) | 5.17 ± 0.65 | 4.67 ± 0.22 |  |  |  |  |  |  |
|  | **Crb** | Female | Tat(−) | 3.75 ± 0.22 | 3.82 ± 0.32 | **9.67** | **0.004** | 0.01 | 0.944 | 1.29 | 0.265 |
|  |  |  | Tat(+) | 3.62 ± 0.19 | 3.62 ± 0.13 |  |  |  |  |  |  |
|  |  | Male | Tat(−) | 4.18 ± 0.20 | 4.00 ± 0.20 |  |  |  |  |  |  |
|  |  |  | Tat(+) | 4.11 ± 0.17 | 4.44 ± 0.24 |  |  |  |  |  |  |
|  | **SC** | Female | Tat(−) | 2.91 ± 0.24 | 3.13 ± 0.23 | 0.24 | 0.625 | **7.97** | **0.008** | 0.29 | 0.595 |
|  |  |  | Tat(+) | 2.35 ± 0.09 | 2.50 ± 0.26 |  |  |  |  |  |  |
|  |  | Male | Tat(−) | 2.97 ± 0.43 | 2.71 ± 0.19 |  |  |  |  |  |  |
|  |  |  | Tat(+) | 2.54 ± 0.24 | 2.33 ± 0.19 |  |  |  |  |  |  |
| **OEA** | **PFC** | Female | Tat(−) | 6.07 ± 0.66 | 5.17 ± 0.17 | 0.33 | 0.573 | 0.00 | 0.953 | 1.03 | 0.319 |
|  |  |  | Tat(+) | 5.74 ± 0.46 | 6.34 ± 0.58 |  |  |  |  |  |  |
|  |  | Male | Tat(−) | 6.11 ± 1.03 | 6.53 ± 0.58 |  |  |  |  |  |  |
|  |  |  | Tat(+) | 5.39 ± 0.66 | 6.30 ± 0.52 |  |  |  |  |  |  |
|  | **Str** | Female | Tat(−) | 3.07 ± 0.13 | 3.08 ± 0.14 | **15.60** | **< 0.001** | 1.98 | 0.169 | 2.29 | 0.140 |
|  |  |  | Tat(+) | 2.88 ± 0.15 | 3.24 ± 0.19 |  |  |  |  |  |  |
|  |  | Male | Tat(−) | 3.40 ± 0.09 | 3.43 ± 0.10 |  |  |  |  |  |  |
|  |  |  | Tat(+) | 4.06 ± 0.51 | 3.60 ± 0.16 |  |  |  |  |  |  |
|  | **Crb** | Female | Tat(−) | 3.07 ± 0.16 | 3.19 ± 0.25 | **5.72** | **0.023** | 0.01 | 0.938 | 1.40 | 0.246 |
|  |  |  | Tat(+) | 2.95 ± 0.13 | 3.03 ± 0.07 |  |  |  |  |  |  |
|  |  | Male | Tat(−) | 3.33 ± 0.13 | 3.21 ± 0.16 |  |  |  |  |  |  |
|  |  |  | Tat(+) | 3.31 ± 0.19 | 3.49 ± 0.17 |  |  |  |  |  |  |
|  | **SC** | Female | Tat(−) | 2.28 ± 0.15 | 2.43 ± 0.15 | 0.01 | 0.908 | **11.38** | **0.002** | 0.42 | 0.524 |
|  |  |  | Tat(+) | 1.86 ± 0.07 | 1.88 ± 0.12 |  |  |  |  |  |  |
|  |  | Male | Tat(−) | 2.33 ± 0.31 | 2.19 ± 0.13 |  |  |  |  |  |  |
|  |  |  | Tat(+) | 1.95 ± 0.15 | 1.91 ± 0.17 |  |  |  |  |  |  |

^a^Levels of PEA and OEA in the prefrontal cortex (PFC), striatum (Str), cerebellum (Crb), and spinal cord (SC) of Tat(−) and Tat(+) female and male mice exposed to acute 30 mg/kg CBD or vehicle expressed as mean ± SEM in nmol/g. A three-way multivariate analysis of variance (MANOVA) for each lipid molecule was conducted with acute CBD, sex, and genotype as between-subjects factors. No effects or interactions were noted for acute CBD administration on any measure and are thus not shown in this table. F-values and p-values are presented from MANOVA results. Bolded values denote significant differences at p ≤ 0.05; mean ± SEM, *n* = 4-5 mice per group.

**Supplementary Figures**

**Western Blot Gels – Raw Images**

**Figure S1. Original and unedited blots of CB_1_R and CB_2_R expression levels in the striatum.** Images show original CB_1_R, CB_2_R, and GAPDH Western blot gels for female and male Tat transgenic mice used for **Figure 6A,B**. For all Western blot gels lane 1 is a control sample represented on all blots. Female Tat(−) control mice treated with vehicle (*n* = 5) are represented by lanes 2-6 and female Tat(−) mice treated with CBD (*n* = 5) are represented by lanes 7-11. Female Tat(+) mice treated with vehicle (*n* = 5) are represented by lanes 13-17 and female Tat(+) mice treated with CBD (*n* = 5) are represented by lanes 18-22. Male Tat(−) control mice treated with vehicle (*n* = 5) are represented by lanes 2-6 and male Tat(−) mice treated with CBD (*n* = 5) are represented by lanes 7-11. Male Tat(+) mice treated with vehicle (*n* = 4) are represented by lanes 13-16 and male Tat(+) mice treated with CBD (*n* = 5) are represented by lanes 17-21. M: molecular weights of marker protein (kDa).

**Figure S2. Original and unedited blots of GPR55 expression levels in the striatum.** Images show original GPR55 and GAPDH Western blot gels for female and male Tat transgenic mice used for **Figure 6C**. For all Western blot gels lane 1 is a control sample represented on all blots. Female Tat(−) control mice treated with vehicle (*n* = 5) are represented by lanes 2-6 and female Tat(−) mice treated with CBD (*n* = 5) are represented by lanes 7-11. Female Tat(+) mice treated with vehicle (*n* = 5) are represented by lanes 13-17 and female Tat(+) mice treated with CBD (*n* = 5) are represented by lanes 18-22. Male Tat(−) control mice treated with vehicle (*n* = 5) are represented by lanes 2-6 and male Tat(−) mice treated with CBD (*n* = 5) are represented by lanes 7-11. Male Tat(+) mice treated with vehicle (*n* = 4) are represented by lanes 13-16 and male Tat(+) mice treated with CBD (*n* = 5) are represented by lanes 17-21. M: molecular weights of marker protein (kDa).

**Figure S3. Original and unedited blots of MAGL and FAAH expression levels in the striatum.** Images show original MAGL, FAAH, and GAPDH Western blot gels for female and male Tat transgenic mice used for **Figure 6D,E**. For all Western blot gels lane 1 is a control sample represented on all blots. Female Tat(−) control mice treated with vehicle (*n* = 5) are represented by lanes 2-6 and female Tat(−) mice treated with CBD (*n* = 5) are represented by lanes 7-11. Female Tat(+) mice treated with vehicle (*n* = 5) are represented by lanes 13-17 and female Tat(+) mice treated with CBD (*n* = 5) are represented by lanes 18-22. Male Tat(−) control mice treated with vehicle (*n* = 5) are represented by lanes 2-6 and male Tat(−) mice treated with CBD (*n* = 5) are represented by lanes 7-11. Male Tat(+) mice treated with vehicle (*n* = 4) are represented by lanes 13-16 and male Tat(+) mice treated with CBD (*n* = 5) are represented by lanes 17-21. M: molecular weights of marker protein (kDa).

**Figure S4. Original and unedited blots of CB_1_R and CB_2_R expression levels in the cerebellum.** Images show original CB_1_R, CB_2_R, and GAPDH Western blot gels for female and male Tat transgenic mice used for **Figure 7A,B**. For all Western blot gels lane 1 is a control sample represented on all blots. Female Tat(−) control mice treated with vehicle (*n* = 5) are represented by lanes 2-6 and female Tat(−) mice treated with CBD (*n* = 5) are represented by lanes 7-11. Female Tat(+) mice treated with vehicle (*n* = 5) are represented by lanes 13-17 and female Tat(+) mice treated with CBD (*n* = 5) are represented by lanes 18-22. Male Tat(−) control mice treated with vehicle (*n* = 5) are represented by lanes 2-6 and male Tat(−) mice treated with CBD (*n* = 5) are represented by lanes 7-11. Male Tat(+) mice treated with vehicle (*n* = 4) are represented by lanes 13-16 and male Tat(+) mice treated with CBD (*n* = 5) are represented by lanes 17-21. The molecular weights of marker protein (kDa) are missing in the blot for female mice. M: molecular weights of marker protein (kDa).

**Figure S5. Original and unedited blots of GPR55 expression levels in the cerebellum.** Images show original GPR55 and GAPDH Western blot gels for female and male Tat transgenic mice used for **Figure 7C**. For all Western blot gels lane 1 is a control sample represented on all blots. Female Tat(−) control mice treated with vehicle (*n* = 5) are represented by lanes 2-6 and female Tat(−) mice treated with CBD (*n* = 5) are represented by lanes 7-11. Female Tat(+) mice treated with vehicle (*n* = 5) are represented by lanes 13-17 and female Tat(+) mice treated with CBD (*n* = 5) are represented by lanes 18-22. Male Tat(−) control mice treated with vehicle (*n* = 5) are represented by lanes 2-6 and male Tat(−) mice treated with CBD (*n* = 5) are represented by lanes 7-11. Male Tat(+) mice treated with vehicle (*n* = 4) are represented by lanes 13-16 and male Tat(+) mice treated with CBD (*n* = 5) are represented by lanes 17-21. M: molecular weights of marker protein (kDa).

**Figure S6. Original and unedited blots of MAGL and FAAH expression levels in the cerebellum.** Images show original MAGL, FAAH, and GAPDH Western blot gels for female and male Tat transgenic mice used for **Figure 7D,E**. For all Western blot gels lane 1 is a control sample represented on all blots. Female Tat(−) control mice treated with vehicle (*n* = 5) are represented by lanes 2-6 and female Tat(−) mice treated with CBD (*n* = 5) are represented by lanes 7-11. Female Tat(+) mice treated with vehicle (*n* = 5) are represented by lanes 13-17 and female Tat(+) mice treated with CBD (*n* = 5) are represented by lanes 18-22. Male Tat(−) control mice treated with vehicle (*n* = 5) are represented by lanes 2-6 and male Tat(−) mice treated with CBD (*n* = 5) are represented by lanes 7-11. Male Tat(+) mice treated with vehicle (*n* = 4) are represented by lanes 13-16 and male Tat(+) mice treated with CBD (*n* = 5) are represented by lanes 17-21. M: molecular weights of marker protein (kDa).

References

1. Dempsey, S. K.; Gesseck, A. M.; Ahmad, A.; Daneva, Z.; Ritter, J. K.; Poklis, J. L., Formation of HETE-EAs and dihydroxy derivatives in mouse kidney tissue and analysis by high-performance liquid chromatography tandem mass spectrometry. J. Chromatogr. B Analyt. Technol. Biomed. Life Sci. 2019, 1126-1127, 121748, DOI:10.1016/j.jchromb.2019.121748.

2.	League, A. F.; Gorman, B. L.; Hermes, D. J.; Johnson, C. T.; Jacobs, I. R.; Yadav-Samudrala, B. J.; Poklis, J. L.; Niphakis, M. J.; Cravatt, B. F.; Lichtman, A. H.; Ignatowska-Jankowska, B. M.; Fitting, S., Monoacylglycerol Lipase Inhibitor MJN110 Reduces Neuronal Hyperexcitability, Restores Dendritic Arborization Complexity, and Regulates Reward-Related Behavior in Presence of HIV-1 Tat. Front Neurol 2021, 12, 651272, DOI:10.3389/fneur.2021.651272.
